# RNA m6A demethylase FTO-mediated epigenetic up-regulation of LINC00022 promotes tumorigenesis in esophageal squamous cell carcinoma

**DOI:** 10.1186/s13046-021-02096-1

**Published:** 2021-09-20

**Authors:** Yuanbo Cui, Chunyan Zhang, Shanshan Ma, Zhe Li, Wenjie Wang, Ya Li, Yingchao Ma, Jiarui Fang, Yaping Wang, Wei Cao, Fangxia Guan

**Affiliations:** 1 School of Life Sciences, Zhengzhou University, Zhengzhou, 450001 China; 2 Translational Medicine Center, Zhengzhou Central Hospital Affiliated to Zhengzhou University, Zhengzhou, 450007 China; 3 Department of Clinical Laboratory, Zhengzhou Central Hospital Affiliated to Zhengzhou University, Zhengzhou, 450007 China; 4 Henan Diagnostic Reagents of Tumor Pathology Research Center, Zhengzhou, 450007 China

**Keywords:** N6-methyladenosine, FTO, LINC00022, Esophageal squamous cell carcinoma, Tumorigenesis, Cell cycle

## Abstract

**Background:**

Long non-coding RNA (LncRNA) controls cell proliferation and plays a significant role in the initiation and progression of esophageal squamous cell carcinoma (ESCC). N6-methyladenosine (m6A) modification now is recognized as a master driver of RNA function to maintain homeostasis in cancer cells. However, how m6A regulates LncRNA function and its role in tumorigenesis of ESCC remain unclear.

**Methods:**

Multiple ESCC datasets were used to analyze gene expression in tumor tissues and normal tissues. Kaplan-Meier method and the ROC curve were conducted to evaluate the prognostic value and diagnostic value of LINC00022 in ESCC, respectively. Both gain-of-function and loss-of-function experiments were employed to investigate the effects of LINC00022 on ESCC growth in vitro and in vivo. Bioinformatics analysis, colorimetric m6A assay, RIP, MeRIP and co-IP was performed to explore the epigenetic mechanism of LINC00022 up-regulation in ESCC.

**Results:**

Here we report that m6A demethylation of LncRNA LINC00022 by fat mass and obesity-associated protein (FTO) promotes tumor growth of ESCC in vivo. Clinically, we revealed that LINC00022 was up-regulated in primary ESCC samples and was predictive of poor clinical outcome for ESCC patients. Mechanistically, LINC00022 directly binds to p21 protein and promotes its ubiquitination-mediated degradation, thereby facilitating cell-cycle progression and proliferation. Further, the elevated FTO in ESCC decreased m6A methylation of LINC00022 transcript, leading to the inhibition of LINC00022 decay via the m6A reader YTHDF2. Over-expression of FTO was shown to drive LINC00022-dependent cell proliferation and tumor growth of ESCC.

**Conclusions:**

Thus, this study demonstrated m6A-mediated epigenetic modification of LncRNA contributes to the tumorigenesis in ESCC and LINC00022, specific target of m6A, serves as a potential biomarker for this malignancy.

**Supplementary Information:**

The online version contains supplementary material available at 10.1186/s13046-021-02096-1.

## Background

Esophageal cancer (ESCA) is one of the most fatal malignancies around the world and thus constitutes a serious challenge to global human health [1]. ESCA consists of two major pathological subtypes, namely esophageal squamous cell carcinoma (ESCC) and esophageal adenocarcinoma (EAC), which differ distinctly in geography and epidemiology [1, 2]. East Asia shows the highest incidence of ESCA, in part due to the incidence in China [3]. ESCC accounts for more than 90% of all Chinese ESCA patients [4]. The global incidence of ESCA gradually declined from 7th in 2012 to 10th in 2020 because of the dietary improvements and medical advances [1, 5]. However, the annual global mortality rate of ESCA remained the sixth in cancer-induced death in the past decade [1, 5]. The 5-year survival rate for patients with advanced ESCA remains dismal, due to uncontrolled tumor growth and relapse and unknown molecular mechanisms [6–8]. In the past few decades, considerable efforts have been made to investigate the molecular mechanisms underlying ESCC pathogenesis [9, 10]. Both genetic, including mutations in tumor suppressor genes such as TP53 and NFE2L2 [11, 12], and epigenetic alterations, including DNA methylation and non-coding RNAs [13–15], have been uncovered to play pivotal roles in the tumorigenesis and development of ESCC. Multi-faced imbalance of gene expression, such as proto-oncogene amplification, mutations, deletions, and epigenetic modifications, could work together to maintain the malignant biological characteristics of ESCC [12, 16]. However, the understanding of oncogenesis of ESCC remains elusive, thus limiting the effective drug development for targeted therapy.

N6-methyladenosine (m6A) is a ubiquitous chemical modification that occurs in the high abundance in eukaryotic cells, and is regulated by a set of specific methyltransferases (writers), demethylases (erasers) and readers [17]. By orchestrating the modification level of m6A on mRNA molecules, the maladjusted m6A regulator affects the stability and translation efficiency of mRNA, and the associated tumor-related biological processes, such as cell proliferation and metastasis [18–20]. Moreover, the function of m6A regulator and its mediated RNA m6A modification level in human cancers is background-dependent. For instance, over-expression of METTL14 decreases the metastatic ability of hepatocellular carcinoma cells through mediating primary microRNA processing [21]. In pancreatic cancer, up-regulated METTL14 stimulates tumor growth and metastasis by increasing PERP translation [22]. These pioneering studies indicate that, similar to other epigenetic modifications such as DNA methylation and histone acetylation, m6A provides an epi-transcriptomic layer for controlling RNA fate and gene expression at the post-transcriptional level. However, the role and mechanism of m6A in ESCC has not been well characterized.

Increasing evidence shows that LncRNAs play crucial roles in remodeling cell viability and genome stability by gene expression regulation [23, 24]. Further in-depth studies demonstrated that LncRNAs regulate tumorigenesis and tumor immunology through direct interaction with endogenous biomolecules such as mRNA, microRNA and proteins [25, 26]. The condition-specific and tissue-specific expression modes of LncRNAs indicate their great potential as clinical targets and biomarkers for tumors [23, 27]. ESCC-related LncRNAs have been recently identified, such as CCAT1 [28], TTN-AS1 [29], LINC01503 [30] and ESCCAL-1 [31], but the role and molecular regulatory mechanism of LncRNAs in ESCC remain elusive. In particular, it is unknown whether m6A regulates LncRNA in ESCC, and how cross-talk of m6A/LncRNA is involved in ESCC tumogenesis.

Herein, we characterized functional role of a novel LncRNA LINC00022 in promoting ESCC growth both in vitro and in vivo. We revealed that it enhances the instability of the cyclin-dependent kinase inhibitor (CDKI) p21 via direct RNA-protein binding. The RNA m6A demethylase FTO was shown to epigenetically regulate the elevation of LINC00022 in ESCC in an YTHDF2-dependent manner. To this end, FTO coordinates with LINC00022 to drive the tumorigenesis of ESCC, thus providing a rationale to target the FTO/LINC00022 axis as a novel therapeutic strategy.

## Materials and methods

### In silico analysis for LINC00022 expression

LncRNA expression datasets from two independent ESCC cohorts, GSE75241 and TCGA-ESCC used as discovery datasets, were retrieved from the Gene Expression Omnibus (GEO) and The Cancer Genome Atlas (TCGA) databases, respectively. GSE75241 contains 15 pairs of ESCC tumor tissues and normal tissues. TCGA-ESCC has 81 ESCC tumor samples and 11 normal samples. Differentially expressed LncRNAs (DELncRNAs) profiles of GSE75241 and TCGA-ESCC were analyzed by using Limma [32] and DESeq2 [33], respectively. LINC00022 expression data in another two ESCC cohorts obtained from the public databases GEPIA [34] and CRN (http://syslab4.nchu.edu.tw) was used as validation datasets.

### Human ESCC tissue specimens

The ESCC cohort in this study included forty-four pairs of surgically resected ESCC tissue specimens from Linzhou Cancer Hospital, Henan province of China were collected and performed to detect the differential gene expression. Thirty-six pairs of ESCC specimens with relatively complete clinical data were utilized to analyze the clinical relevance of gene expression. All patients signed informed consent and did not receive any chemotherapy or radiotherapy before surgery. The protocols were approved by the Ethical Review Committee of Zhengzhou University.

### Receiver operating character curve

The diagnostic value of LINC00022 in ESCC was analyzed by receiver operating character (ROC) curve based on the expression level of LINC00022 in tumor tissues and normal tissues in each ESCC cohort. The sensitivity is represented by the area under the curve (AUC), and the higher the AUC, the greater the diagnostic value.

### Secondary structure prediction for LINC00022

The secondary structure of the full-length sequence of LINC00022 transcript at minimum free energy (MFE) was computerized by the RNAfold web server (http://rna.tbi.univie.ac.at//cgi-bin/RNAWebSuite/RNAfold.cgi).

### RNA-protein interaction prediction

The potential RNA-protein interactions between LINC00022 and p16, p21, p53 were predicted by the machine learning classifier RPISeq [35] based on Random Forest (RF) or Support Vector Machine (SVM) classifiers.

### Kyoto encyclopedia of genes and genomes (KEGG) pathway

The putative biological function of LINC00022 in ESCC was analyzed by gene enrichment method. We first obtained a thousand LINC00022-associated genes in ESCC tissues from GEPIA database, and then used WebGestalt (http://www.webgestalt.org/) to analyze the clustering properties of these genes based on the KEGG pathway. The result of pathway enrichment analysis is shown as a bubble diagram.

### Copy number variation and DNA methylation analysis

The genome copy number variation (CNV) data of LINC00022 was obtained from TCGA and analyzed by GISTIC2.0 [36]. The correlation between LINC00022 expression and CNV level of LINC00022 in 159 cases of esophageal cancer tissues was analyzed by Person’s Coefficient. DNA methylation data of LINC00022 promoter was also retrieved from the TCGA project and analyzed by Limma package. The correlation between LINC00022 expression and methylation level of LINC00022 promoter in 161 cases of esophageal cancer tissues was then analyzed.

### Cell culture

Human ESCC cell lines KYSE150 and TE1 were purchased from Procell (Wuhan, China). KYSE70, KYSE450 and one human esophageal epithelial cell line Het-1A were kindly provided by the Bioengineering and Transformation Laboratory of Zhengzhou University. All cell lines were identified by short tandem repeat (STR) analysis and cultured in RPMI 1640 (Procell, China) basic medium supplemented with 1% penicillin-streptomycin solution (Procell, China) and 10% fetal bovine serum (FBS) (LONSERA, Uruguay) at 37 °C containing 5% CO_2_.

### Plasmids and siRNAs transfection

The recombinant plasmids for over-expressing YTHDF2 (OE-YTHDF2) and the negative control plasmids (OE-NC) were purchased from GeneChem (Shanghai, China). Three small interfering RNAs (siRNAs) against LINC00022 (si-022#1, si-022#2, si-022#3) and the negative control siRNAs (si-NC) were purchased from Public Protein/Plasmid Library (PPL) (Nanjing, China). The siRNAs targeting YTHDF2 (si-YTHDF2) were synthesized by GenePharma (Shanghai, China). Lipofectamine 3000 (Invitrogen, USA) and INTERFERin (Polyplus, France) were used for transfecting plasmids and siRNAs into cells, respectively.

### Recombinant lentivirus and infection

Recombinant lentiviruses for knocking down LINC00022 or FTO (sh-LINC00022 or sh-FTO) and lentiviruses for over-expressing LINC00022 or FTO (OE-LINC00022 or OE-FTO) as well as the corresponding control viruses were purchased from GeneChem (Shanghai, China). HiTransG A or HiTransG P solution (GeneChem, China) was used to increase the efficiency of cell infection.

### RNA isolation and qRT-PCR

Total RNAs from cells or tissues were extracted by Trizol (Invitrogen, USA) reagent and quantified by Nanodrop 2000 (Thermo, USA). After cDNA synthesis with the NovoScript All-in-one SuperMix (Novoprotein, China), the 7500 Fast Real-Time System (AB, USA) and NovoStart SYBR SuperMix (Novoprotein, China) were employed to perform the qRT-PCR assay. The house-keeping gene GAPDH was used as an internal reference. The qRT-PCR primer sequences used in this study are shown in Supplementary Table 1.

### Cell proliferation assays

Cell proliferation was evaluated by the CCK-8, EdU staining and colony formation assays. For the CCK-8 assay, cells were inoculated in 96-well plates and maintained in an incubator for a specified period of time. 10 μL of CCK-8 solution (7sea biotech, China) was added to each well 3 h prior to measurement at 450 nm. For the EdU staining assay, cells were labeled with EdU staining solution (Abbkine, USA) at 37 °C for 4 h, fixed in 4% formaldehyde and permeabilized with 0.5% Triton X-100. Then the cells were incubated in Click-iT mixture and re-stained with DAPI solution, and finally detected under a fluorescent microscope. For the colony formation assay, cells were seeded in 12-well plates and maintained in an incubator for 8 to 12 days to form the colonies. After fixation and crystal violet staining, the colonies in each well were photographed and then counted by the Image J software.

### Flow cytometry analysis

The Annexin V-FITC/PI double staining kit (7sea biotech, China) and PI staining kit (7sea biotech, China) were used for testing cell apoptosis and cell cycle, respectively. Cells stained with Annexin V-FITC and PI solution in turn were subjected to evaluate apoptotic rates by a flow cytometer (Beckman, USA). Cells fixed in 70% ethanol and incubated with PI solution containing RNase were used to analyze cell-cycle changes.

### Western blot

Total proteins were isolated from cells or tissues using the RIPA lysis buffer (Beyotime, China) and subjected to 10% polyacrylamide gel electrophoresis. After transferring to PVDF membrane, blocking with 5% skim milk, incubation with primary antibodies and secondary antibodies, the proteins on the membrane were finally detected by ECL kit (EpiZyme, China) and imaging analysis system (BioRad, USA). The primary antibodies used in this study were as follows: anti-GAPDH (Bioworld, 1:10000), anti-CDK2 (Bioworld, 1:1000), anti-Cyclin E1 (Bioworld, 1:1000), anti-CDK4 (Bioworld, 1:1000), anti-Cyclin D1 (Bioworld, 1:1000), anti-p53 (Bioss, 1:500), anti-p21 (Bioworld, 1:500), anti-p16 (Bioss, 1:1000), anti-ubiquitin (Santa, 1:500), anti-FTO (Bioss, 1:1000), anti-YTHDF2 (Abcam, 1:1000).

### Protein stability analysis

The transfected cells were inoculated in 6-well plates and maintained in an incubator overnight at 37 °C. Then the cells were treated with cycloheximide (CHX) (Sigma, USA) at a concentration of 100 μg/mL for the indicated times. Total proteins in each group were extracted and subjected to Western blot analysis.

### RNA-protein immunoprecipitation (RIP) analysis

The Magna RIP Kit (Millipore, USA) combined with qRT-PCR were utilized to analyze the direct interactions between LINC00022 transcript and indicated proteins. The procedures were carried out according to users’ instructions of the kit. Cells were broken up with RIP lysis buffer containing protease inhibitor cocktail and RNase inhibitors. Then the cell lysates and magnetic beads conjugated with specific antibodies or IgG were incubated in RIP immunoprecipitation buffer at 4 °C for 3 h with rotation. After digestion with protease K, the immunoprecipitated RNA samples were purified by phenol/chloroform/isoamyl alcohol and 100% ethanol, and finally detected by qRT-PCR and 1.5% agarose gel electrophoresis.

### Co-immunoprecipitation (co-IP)

The Pierce Classic Magnetic Co-IP Kit (Thermo, USA) combined with Western blot was utilized to detect the effect of LINC00022 over-expression on ubiquitination of p21 in ESCC cells. In brief, cells were broken up with ice-cold IP lysis buffer to obtain the supernatant containing total proteins. The cell lysates were incubated with antibodies in a tube overnight at 4 °C to form the immunoprecipitation complex. Then the pre-treated protein A/G beads were added to each tube and incubated at room temperature for 1 h. Finally, the supernatant containing the target antigen collected by the magnetic stand was used for Western blot analysis.

### N6-methyladinosine modification prediction

Two reliable online tools RMBase v2.0 [37] and SRAMP [38], based on epitranscriptome sequencing information and machine learning pattern, were used to predict potential m6A modification sites on the LINC00022 transcript. The m6A2Target (http://m6a2target.canceromics.org) database was employed to predict m6A modification enzymes that may perturb LINC00022 expression based on MeRIP-seq or RNA-seq profiles.

### Total RNA m6A quantification

The overall RNA m6A level was measured by the EpiQuik m6A RNA Methylation Kit (Colorimetric) (Epigentek, USA). In brief, total RNAs were isolated from cells or tissues and quantified by Nanodrop 2000 (Thermo, USA). Total RNA of 200 ng and binding solution were added to each well and incubated at 37 °C for 1.5 h. Then the capture antibody, detection antibody, enhancer solution, developer solution and stop solution were added to each well in turn and reacted for the indicated time. Finally, the relative m6A level in each group was compared by detecting the absorbance value at 450 nm via a microplate reader (Molecular Devices, USA).

### Methylated RNA immunoprecipitation (MeRIP) assay

The Magna MeRIP m6A Kit (Millipore, USA) was utilized to determine the enrichment of m6A at specific sites on the LINC00022 transcript. The fragmentation buffer was first used to fragment 300 μg of total RNA (at a concentration of 1 μg per μL) in each sample. Then the fragmented RNA and magnetic beads conjugated with m6A or IgG antibodies were incubated in the MeRIP reaction mixture at 4 °C for 2 h with rotation. Finally, the co-precipitated RNA samples containing m6A modification sites were used for qRT-PCR detection. Three specific primers were designed for MeRIP-qRT-PCR according to the 3 m6A modification sites on LINC00022 transcript with higher confidence predicted by RMBase v2.0 and SRAMP (Supplementary Fig. 10A-B). The primer sequences are listed in Supplementary Table 1.

### RNA stability analysis

The transfected cells were seeded in 12-well plates and maintained in an incubator overnight at 37 °C. Then the cells were treated with Actinomycin D (ActD) (Sigma, USA) at a concentration of 5 μg/mL for the indicated times. Total RNAs in each group were extracted and subjected to qRT-PCR analysis.

### In vivo tumorigenesis

The 6-week-old male BALB/c nude mice were purchased from Beijing Vital River Co., Ltd. (Beijing, China). All the mice were housed in automatic air cages with free access to food and water. For in vivo tumorigenesis experiments, ESCC cells with stable over-expression or knockdown of the target gene were inoculated subcutaneously on the right dorsal side of mice. For Figs. 3A, C, 8H and I, the number of cells inoculated in each mouse was 4 × 10^6^, 1 × 10^6^, 2 × 10^6^ and 1 × 10^6^, respectively. Tumor size was measured using the length × width^2^ × 0.5 formula. At the end of each experiment, the tumors were isolated and weighed. Each tumor was divided into three parts, one for paraffin sectioning and staining (e.g., HE or Ki-67 IHC), one for total RNA extraction, and one for total protein extraction.

### Statistical analysis

All experimental data are displayed as mean ± SD from at least three replicates. SPSS 19.0 and Graphpad 7.0 software were used for statistical analysis and plotting of the data. Student’s t-test was used to compare the differences between the two groups of data. Pearson’s Coefficient was utilized to analyze the expression correlation between two genes. Survival curve and hazardous ratio (HR) for the indicated gene and ESCC cohort were detected by the Kaplan-Meier method and log-rank test. A *p*-value < 0.05 was considered as statistically significant.

## Results

### LINC00022 is highly expressed in ESCC and predicts poor prognosis

**Fig. 1:**
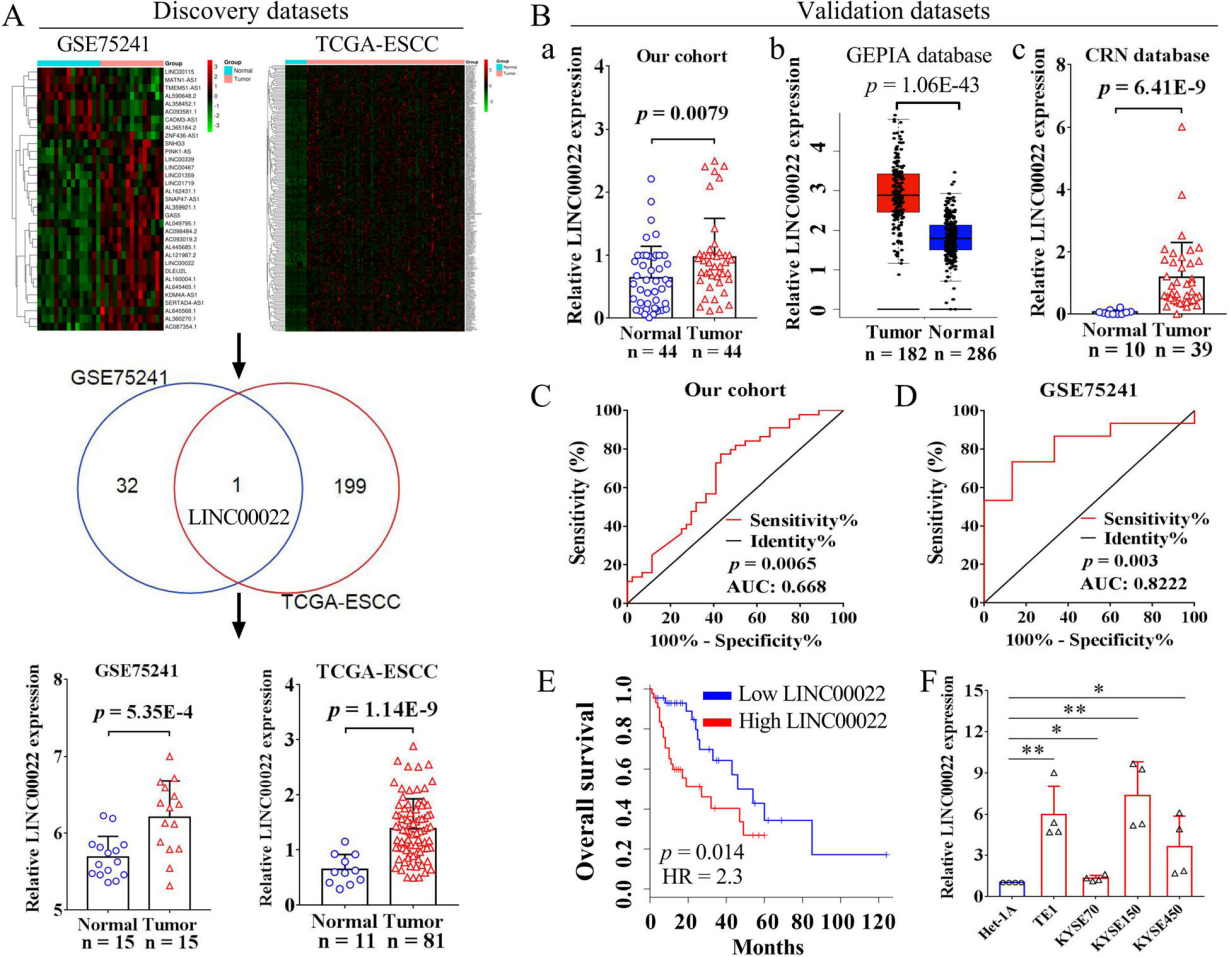
Up-regulated LINC00022 predicts poor prognosis of ESCC patients. (A) LINC00022 was one of the most up-regulated LncRNAs in two independent ESCC cohorts from GSE75241 and TCGA. The overall differentially expressed LncRNAs (DELncRNAs) in GSE75241 (15 normal tissues vs. 15 tumor tissues) dataset were shown as a heat map (A, upper left). The top-200 up-regulated DELncRNAs in TCGA-ESCC (11 normal tissues vs. 81 tumor tissues) dataset were shown as a heat map (A, upper right). The Venn diagram in the middle shows that LINC00022 is the only overlapped LncRNA in the two discovery datasets. Box plots from both GSE75241 and TCGA-ESCC datasets indicate the significant up-regulation of LINC00022 in ESCC tumor samples as compared to normal samples (A, bottom). (B) The elevation of LINC00022 was validated by additional three ESCC cohorts, including our in-house cohort (a, 44 pairs of ESCC tissues), GEPIA cohort (b, 286 normal tissues vs. 182 tumor tissues) and CRN cohort (c, 10 normal tissues vs. 39 tumor tissues). (C-D) The diagnostic values of LINC00022 in our cohort and GSE75241 cohort were evaluated by ROC. (E) The prognostic significance of LINC00022 in GEPIA cohort was determined by the Kaplan-Meier method. Increased LINC00022 expression predicted an unfavorable patient OS. (F) The expression of LINC00022 in Het-1A, TE1, KYSE70, KYSE150 and KYSE450 was examined by qRT-PCR, *p < 0.05; **p < 0.01

To identify potential oncogenic LncRNAs in ESCC, we analyzed the differential expression profiles of LncRNAs (DELncRNAs) from two independent ESCC cohorts, GSE75241 and TCGA-ESCC. A total of 33 and 3134 DELncRNAs were analyzed from datasets GSE75241 and TCGA-ESCC, respectively. We compared 33 DELncRNAs from GSE75241 with the top-200 up-regulated DELncRNAs from TCGA-ESCC, and found that LINC00022 was the only LncRNA that was significantly elevated in both datasets (Fig. 1A). LINC00022 expression in ESCC tumor samples and normal samples in datasets GSE75241 (*p* = 5.35E-4) and TCGA-ESCC (*p* = 1.14E-9) was shown as box plots (Fig. 1A). These results implicated that LINC00022 plays an important role in ESCC. We then analyzed the expression of LINC00022 in our in-house cohort containing 44 pairs of ESCC tumor specimens and matched normal specimens by qRT-PCR. In line with the above dataset analysis, LINC00022 was indeed elevated in our cohort ESCC tumors (Fig. 1B-a, *p* = 0.0079). We also analyzed the paired results of LINC00022 expression in these 44 cases of ESCC. As shown in Supplementary Fig. 1A, LINC00022 was up-regulated in 33 ESCC tumors (75%) and down-regulated in 11 ESCC tumors (25%). When interrogating two additional ESCC datasets from GEPIA (Fig. 1B-b, *p* = 1.06E-43) and CRN (Fig. 1B-c, *p* = 6.41E-9) databases, we found that LINC00022 was consistently increased in tumor tissues compared to normal tissues. In addition, LINC00022 was likewise significantly up-regulated in tumor samples from the TCGA-EAC and TCGA-ESCA datasets (Supplementary Fig. 1B-C), implying that it also plays a role in EAC.

To gain a better understanding of the potential role of LINC00022 in human pan-cancer, we used the comprehensive database GEPIA to analyze the expression landscape of LINC00022 in 28 types of cancer other than ESCC. LINC00022 was found highly expressed in tumor tissues of CESC (*p* = 1.16E-6), DLBC (*p* = 2.83E-21), GBM (*p* = 2.31E-35), SKCM (*p* = 7.77E-89), STAD (*p* = 2.74E-62), and THYM (*p* = 6.09E-55), while low expressed in tumor tissues of TGCT (*p* = 4.15E-45), when compared with the corresponding normal tissues (Supplementary Fig. 2A). No significant difference was found as to the expression of LINC00022 between tumor samples and normal samples in ACC, BLCA, BRCA, CHOL, COAD, HNSC, KICH, KIRC, KIRP, LAML, LGG, LIHC, LUAD, LUSC, OV, PAAD, PRAD, READ, THCA, UCEC, and UCS (Supplementary Fig. 2B-D). Kaplan-Meier analysis from GEPIA showed that LINC00022 expression was not associated with OS in patients with CESC, DLBC, GBM, SKCM, STAD, THYM, and TGCT (Supplementary Fig. 3). This indicates that LINC00022 plays a specific role in ESCC, but not in other types of cancer.

We next analyzed the correlation between LINC00022 and clinical parameters using our 36 patients with complete information in our in-house cohort. We found that LINC00022 was up-regulated in tumor tissues of female patients or advanced stage patients, when compared with male or early stage patients (Supplementary Fig. 4A), respectively. In addition, no significant correlation was found between the expression of LINC00022 and age, differentiation, grade, lymph node metastasis or depth of invasion (Supplementary Fig. 4B-C). ROC curve was conducted to evaluate the predictive values of LINC00022 in a variety of ESCC cohorts. The AUC of LINC00022 in our cohort, GSE75241, TCGA-ESCC, and CRN was 0.668 (Fig. 1C, *p* = 0.0065), 0.8222 (Fig. 1D, *p* = 0.003), 0.9001 (Supplementary Fig. 5A, *p* < 0.0001), and 0.9795 (Supplementary Fig. 5B, *p* < 0.0001), respectively. LINC00022 was also showed high diagnostic values in datasets TCGA-EAC (Supplementary Fig. 5A, AUC = 0.9398, *p* < 0.0001) and TCGA-ESCA (Supplementary Fig. 5A, AUC = 0.9198, *p* < 0.0001). Kaplan-Meier analysis of ESCC cohort from GEPIA revealed that high expression of LINC00022 was significantly associated with poor patient OS (Fig. 1E, HR = 2.3, *p* = 0.014) and was a risk factor for unfavorable DFS (Supplementary Fig. 5C, HR = 1.9, *p* = 0.063). Additionally, survival analysis from TCGA cohorts depicted that patients with high LINC00022 expression had a shorter median survival than those with low LINC00022 expression in both ESCC (Supplementary Fig. 5D, HR = 1.99, *p* = 0.17, 25.4 months vs. 42.1 months) and EAC (Supplementary Fig. 5E, HR = 2.35, *p* = 0.012, 16.5 months vs. 46.7 months). Furthermore, LINC00222 was frequently up-regulated in ESCC cell lines as compared to Het-1A (Fig. 1F). Collectively, these data suggest that LINC00022 is highly expressed in ESCC, and is of diagnostic and predictive value.

### LINC00022 facilitates the growth of ESCC cells in vitro

**Fig. 2:**
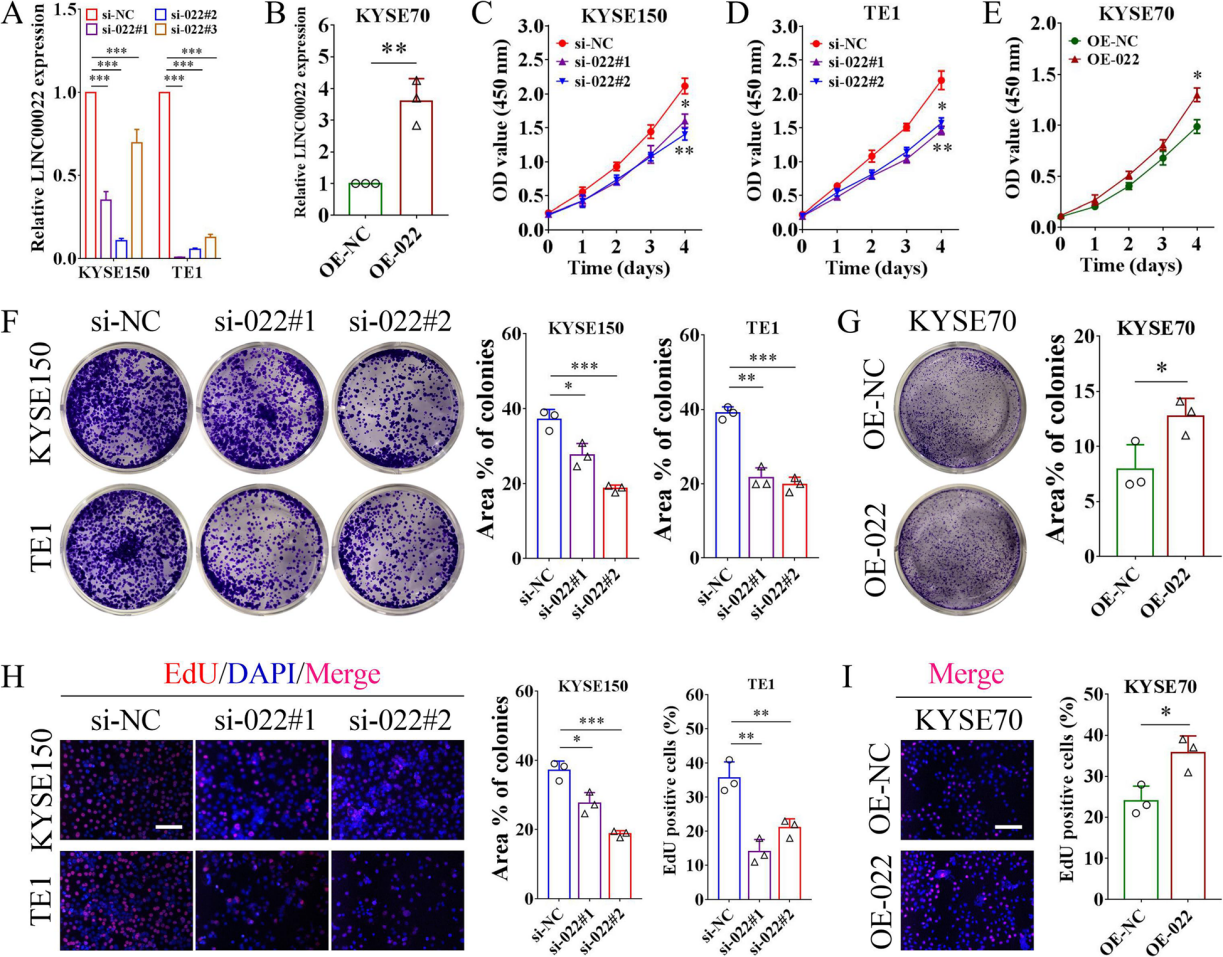
LINC00022 facilitates ESCC proliferation in vitro. (A) The knockdown efficiencies of three specific siRNAs on LINC00022 in KYSE150 and TE1 cells were tested by qRT-PCR, ***p < 0.001. (B) The expression of LINC00022 in KYSE70 cells infected with recombinant over-expressed lentivirus (OE-022) was determined by qRT-PCR, **p < 0.01. (C-E) CCK-8 assay was carried out to evaluate cell viability of KYSE150 and TE1 with LINC00022 knockdown (C, D), and KYSE70 with LINC00022 over-expression (E), *p < 0.05; **p < 0.01. (F-G) Ablation of LINC00022 attenuated the colony formation ability of KYSE150 and TE1 (F), while over-expression of LINC00022 enhanced the colony formation ability of KYSE70 (G), *p < 0.05; **p < 0.01; ***p < 0.001. (H-I) EdU staining assay was conducted to examine cell proliferation of KYSE150 and TE1 with LINC00022 knockdown (H), and KYSE70 with LINC00022 over-expression (I), *p < 0.05; **p < 0.01. Scale bar = 100 μm

To determine the oncogenic properties of LINC00022 in ESCC, we first performed gene enrichment analysis. To this end, one thousand LINC00022-associated genes were obtained from 182 ESCC sequenced samples in GEPIA database and then subjected to KEGG analysis. The LINC00022-related genes were found to be significantly enriched in biological processes such as DNA replication and cell cycle (Supplementary Fig. 6), suggesting that LINC00022 may play a crucial role in ESCC cell proliferation. We used siRNAs to specifically reduce the intracellular RNA level of LINC00022 (Fig. 2A) and the Lentivirus-mediated system to activate the expression of LINC00022 (Fig. 2B) in ESCC cells. Then, the proliferation ability of ESCC cells following LINC00022 manipulation was evaluated by CCK-8, colony formation and EdU incorporation assays. CCK-8 viability assay demonstrated that silencing of LINC00022 effectively repressed the cell survival of KYSE150 and TE1 (Fig. 2C-D), while over-expression of LINC00022 enhanced the viability of KYSE70 cells (Fig. 2E). In addition, depletion of LINC00022 significantly impaired the colony-forming capacity of ESCC cells (Fig. 2F), and enforced expression of LINC00022 elicited the opposite biological effect (Fig. 2G). Furthermore, EdU staining assay showed that LINC00022 deletion in both KYSE150 and TE1 cells suppressed their growth (Fig. 2H), whereas ectopic expression of LINC00022 markedly promoted the proliferative ability of KYSE70 cells (Fig. 2I). Together, both loss-of-function and gain-of-function experiments illustrate that LINC00022 facilitates cell proliferation of ESCC in vitro.

### LINC00022 drives tumorigenesis of ESCC in vivo

**Fig. 3:**
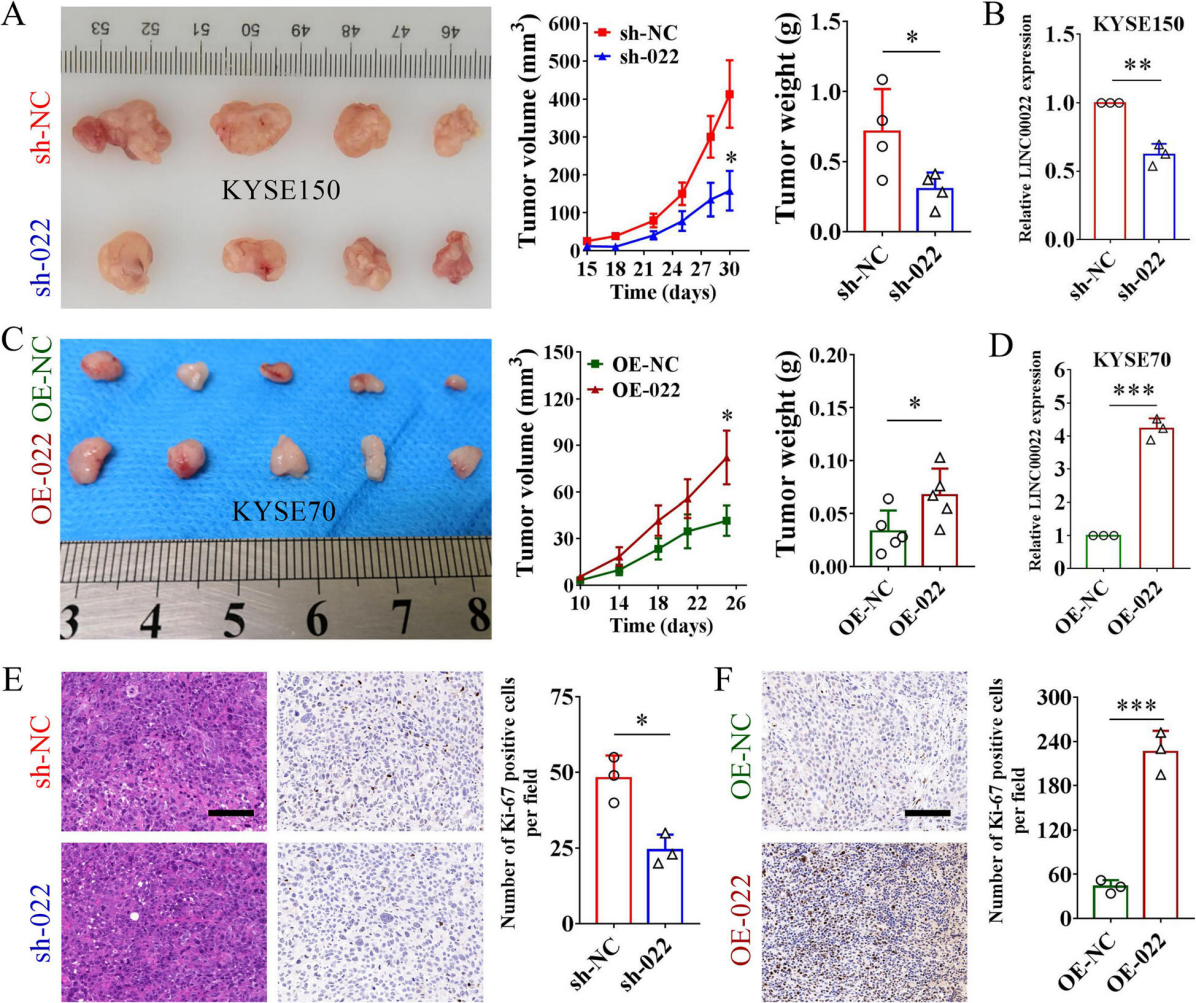
LINC00022 promotes tumorigenesis of ESCC in vivo. (A) Knockdown of LINC00022 effectively attenuated cell growth of KYSE150 in nude mice (*n* = 4) (left panel). The tumor volume was recorded every three days from day 15 to day 30 after cell transplantation, and the curve was plotted (middle panel). Tumors were weighed immediately after removed from the nude mice (right panel), *p < 0.05. (B) The expression of LINC00022 in tumors was analyzed by qRT-PCR, **p < 0.01. (C) Over-expression of LINC00022 evidently promoted subcutaneous tumorigenicity of KYSE70 cells in nude mice (*n* = 5) (left panel). The tumor volume was monitored every four days from day 10 to day 26 after cell inoculation, and the curve was generated (middle panel). Over-expression of LINC00022 resulted in greater tumor size (right panel), *p < 0.05. (D) Analysis of LINC00022 expression in tumors by qRT-PCR, ***p < 0.001. (E) Tumor sections were stained with HE (left panel) and Ki-67 antibody (middle panel) (scale bar = 100 μm). Tumor sections with LINC00022 knockdown had fewer Ki-67 positive cells (right panel), *p < 0.05. (F) The number of Ki-67 positive cells in tumor sections was examined by IHC (left panel). Tumors with LINC00022 over-expression had more Ki-67 positive cells (right panel), ***p < 0.001

Given that LINC00022 is critical for sustaining the proliferation of ESCC cells, we further used the xenograft nude mice model to verify the in vivo role of LINC00022 in ESCC tumorigenesis. KYSE150 cells with Lentivirus-mediated stable knockdown of LINC00022 (sh-022) and KYSE70 cells with stable over-expression of LINC00022 (OE-022) were inoculated subcutaneously into nude mice to investigate the role of LINC00022 on the tumorigenicity of ESCC cells. Notably, LINC00022 knockdown decreased the growth capacity of KYSE150 cells in nude mice, showing smaller tumor volume and lower tumor weight (Fig. 3A). Decreased LINC00022 expression was observed in tumors of sh-022 groups (Fig. 3B). Likewise, over-expression of LINC00022 significantly increased the tumorigenicity of KYSE70 cells in nude mice, as indicated by larger tumors (Fig. 3C) given the elevated expression of LINC00022 in tumors of OE-022 groups detected by qRT-PCR (Fig. 3D). Consistent with these observations, IHC results showed that Ki-67, a molecular marker of cell proliferation, was decreased in LINC00022-deficient tumors (Fig. 3E) and up-regulated in LINC00022-augmented tumors (Fig. 3F) as compared with the corresponding controls. All these data imply that LINC00022 as a driving factor in ESCC tumorigenesis.

### LINC00022 promotes cell-cycle progression in ESCC cells

**Fig. 4:**
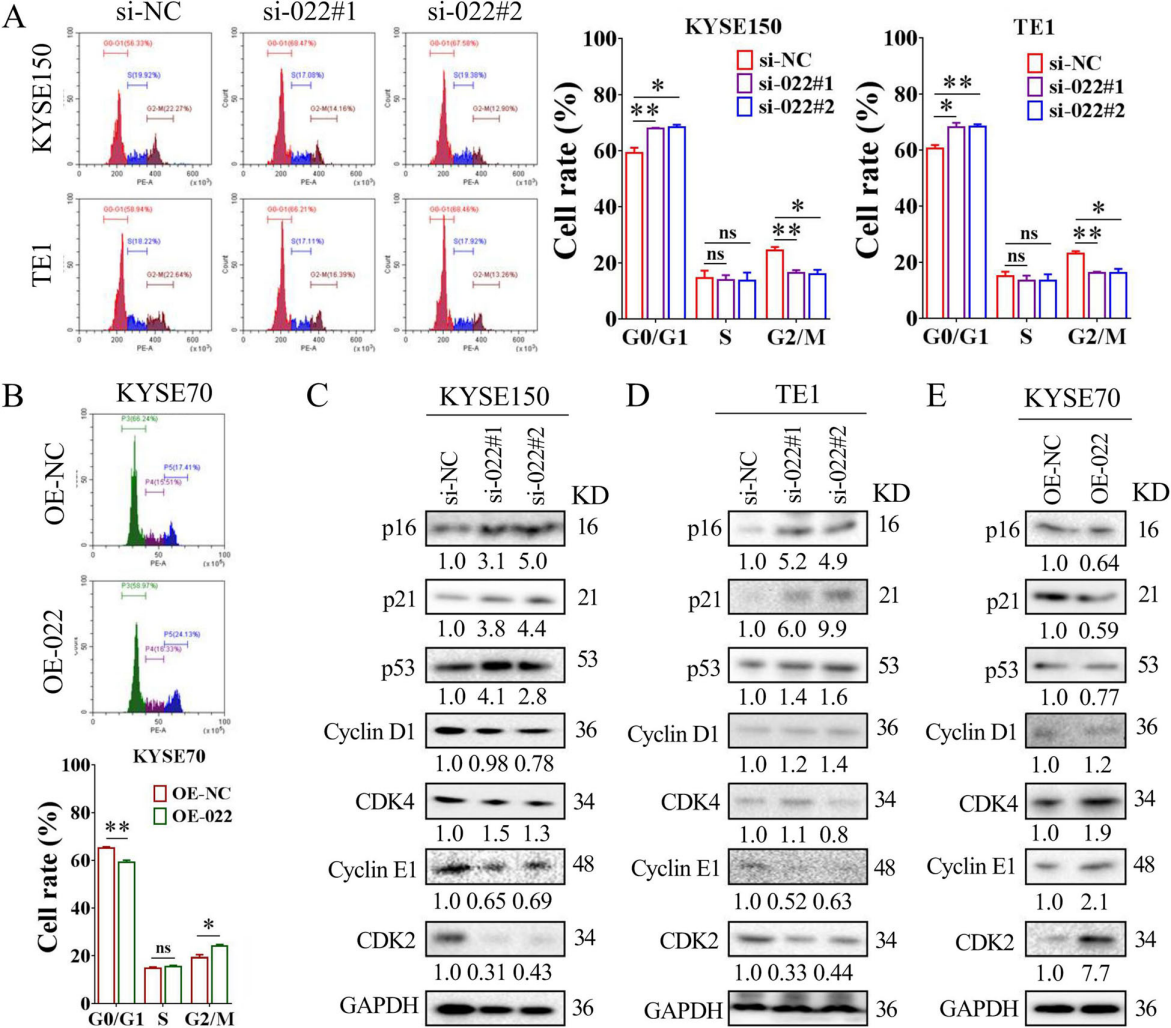
LINC00022 promotes cell-cycle progression in ESCC cells. (A) Cell cycle phase distribution of KYSE150 and TE1 following siRNA-mediated knockdown of LINC00022 was detected by PI-staining flow cytometry (left panel). Ablation of LINC00022 led to a significant increase in the proportion of cells in G0/G1 phase, a slight decrease in the proportion of cells in S phase but with no significant difference, and an obvious decrease in the proportion of cells in G2/M phase (right panel), *p < 0.05; **p < 0.01; ns means no significance. (B) Cell-cycle change of KYSE70 cells after LINC00022 over-expression was evaluated by PI-labeling flow cytometry (upper panel). Up-regulation of LINC00022 resulted in a significant reduction in the proportion of cells in G0/G1 phase, a slight increase in the proportion of cells in S phase but with no significant difference, and a dramatic elevation in the proportion of cells in G2/M phase (nether panel), *p < 0.05; **p < 0.01; ns means no significance. (C-E) Western blot was used to detect the changes of cell cycle regulator protein levels in KYSE150 (C) and TE1 (D) cells following LINC00022 knockdown, and in KYSE70 (E) cells following LINC00022 over-expression. The positive cell-cycle regulators include CDK2, Cyclin E1, CDK4 and Cyclin D1, while the negative regulators include p16, p21 and p53. The numbers below each band represent the relative protein levels change

Accelerated cell-cycle progression is a key hallmark of malignant proliferation of cancer cells [39, 40], and targeted inhibition of cell cycle is widely considered to be an effective clinical anti-tumor strategy [41, 42]. Flow cytometry analysis revealed that siRNA-mediated silencing of LINC00022 induced changes in the cell-cycle distribution of KYSE150 and TE1 cells, resulting in an increased proportion of G0/G1 cells and a decreased ratio of G2/M cells (Fig. 4A). While, the over-expression of LINC00022 led to a decreased G0/G1 phase and prolonged G2/M phase in KYSE70 cells (Fig. 4B). Importantly, LINC00022 knockdown was not significantly associated with apoptosis in both KYSE150 and TE1 cells, as detected by Annexin V-FITC/PI double staining and PI-labeling assays (Supplementary Fig. 7A-B). Additionally, the protein expression of a panel of cell-cycle regulators in ESCC cells following LINC00022 dysregulation was examined by Western blot. Ablation of LINC00022 resulted in consistent up-regulation of CDK inhibitors p16, p21, and p53 in KYSE150 and TE1 cells (Fig. 4C-D), while over-expression of LINC00022 decreased the protein levels of p16, p21 and p53 in KYSE70 cells (Fig. 4E). Concomitantly, the protein levels of CDK2 and Cyclin E1, but not CDK4 and Cyclin D1, in ESCC cells decreased with LINC00022 deletion and increased with LINC00022 over-expression (Fig. 4C-E). These results suggest that LINC00022 regulates cell-cycle progression in ESCC cells.

### LINC00022 de-stabilizes p21 protein via direct RNA-protein binding

**Fig. 5:**
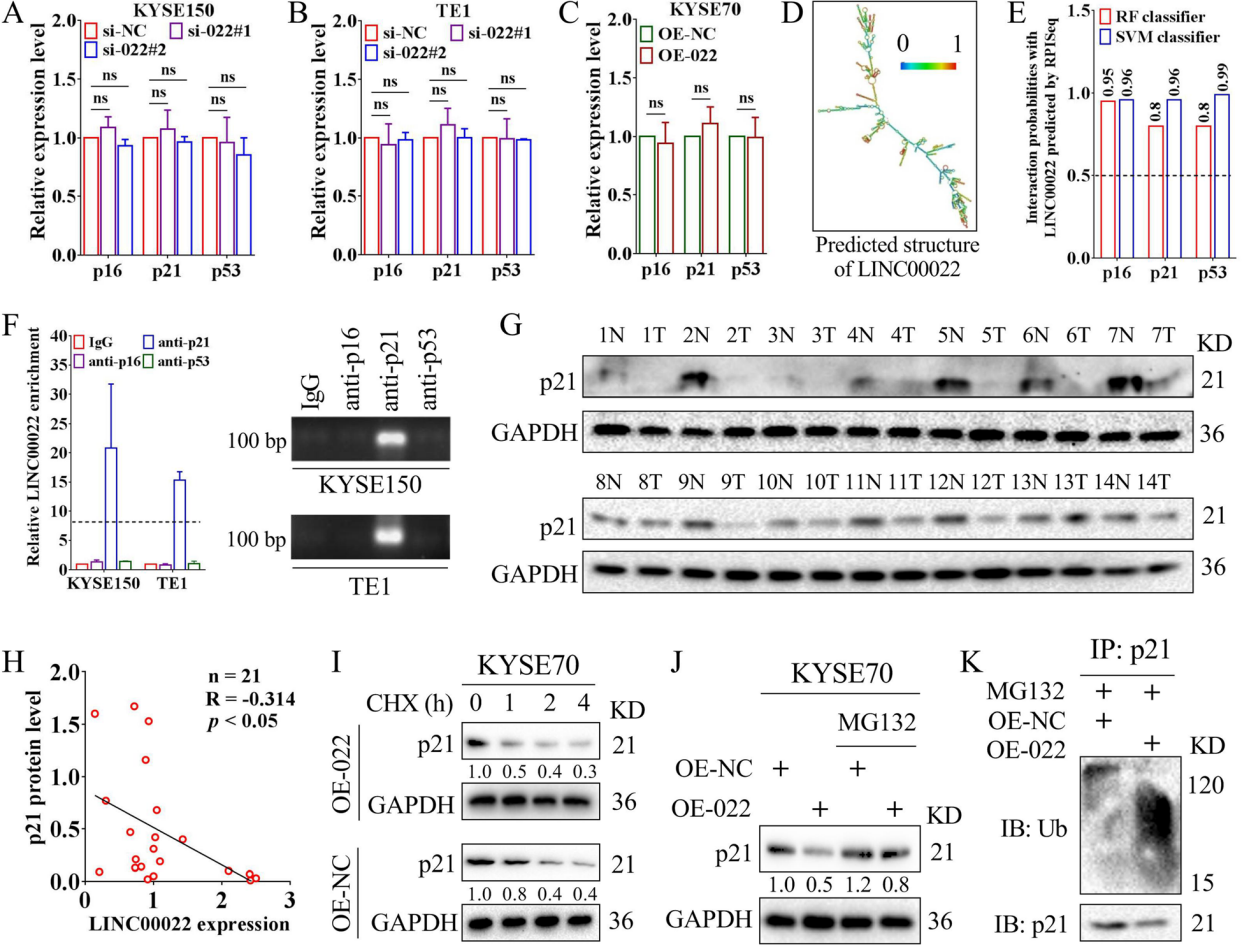
LINC00022 promotes ubiquitin-mediated p21 protein instability. (A-C) The mRNA levels of p16, p21 and p53 in KYSE150 (A) and TE1 (B) cells following LINC00022 knockdown and in KYSE70 (C) cells following LINC00022 over-expression were tested by qRT-PCR, ns means no significance. The mRNA levels of p16, p21 and p53 in ESCC cells were not significantly affected by either LINC00022 knockdown or LINC00022 over-expression. (D) The putative secondary structure of LINC00022 transcript in minimum free energy (MFE) mode was computational analyzed by RNAfold server. (E) The affinity of LINC00022 transcript with p16, p21, and p53 proteins was predicted by the machine learning classifier RPISeq based on Random Forest (RF) and Support Vector Machine (SVM) classifiers. (F) The direct binding affinity of LINC00022 transcript with p16, p21, or p53 proteins was determined by RIP-qRT-PCR (left panel) and agarose gel electrophoresis (right panel). (G) Western blot was used to examine the protein levels of p21 in 21 pairs of ESCC tissues, of which 14 pairs were shown. N represents normal and T represents tumor. (H) Person’s Coefficient analysis revealed the negative correlation between LINC00022 transcription and p21 protein in 21 cases of ESCC tumors. (I) Western blot was performed to observe the effect of LINC00022 over-expression on the stability of p21 protein in ESCC cells in the presence of protein synthesis inhibitor CHX (100 μg/mL). (J) The proteasome inhibitor MG132 (5 μM) partially relieved the instability of p21 protein caused by LINC00022 over-expression. (K) Co-IP combined with Western blot revealed the activated ubiquitination of p21 protein induced by LINC00022 over-expression

Since LINC00022 manipulation affects the protein levels of CDK inhibitors in ESCC cells, we next explored its potential mechanism. Results of qRT-PCR indicated that deletion or over-expression of LINC00022 did not significantly affect the mRNA levels of p16, p21 and p53 in ESCC cells (Fig. 5A-C). The secondary structure analysis based on the minimum free energy (MFE) algorithm of the RNAfold server showed that LINC00022 contained multiple branching structures (Fig. 5D), suggesting that LINC00022 has the protein binding potential. The machine learning classifier RPISeq based on Random Forest (RF) and Support Vector Machine (SVM) classifiers predicted that the LINC00022 transcript had a high affinity with p16, p21, and p53 proteins (Fig. 5E). Binding with functional proteins is a common molecular regulatory mechanism by which LncRNAs exert their biological functions [23, 24]. Subsequent RIP-qRT-PCR assays demonstrated that LINC00022 bound directly with p21 protein, but not with p16 or p53 proteins, in ESCC cells (Fig. 5F). We next performed Western blot to detect p21 protein levels in 21 cases of ESCC (showing 14 cases) and found that p21 was reduced in most tumor samples relative to normal samples (Fig. 5G). In addition, the inverse correlation between LINC00022 transcription and p21 protein in 21 cases of ESCC tumors was analyzed by Person’s Coefficient (Fig. 5H, R = − 0.314), which was consistent with the negative regulation of p21 protein levels by LINC00022 in ESCC cells (Fig. 4C-E). Clinically, Kaplan-Meier analysis of TCGA-ESCC dataset showed that patients with high level of p21 had longer DFS (Supplementary Fig. 8A).

KEGG-based gene enrichment analysis in Supplementary Fig. 6 also showed that LINC00022 may be involved in proteasome and ubiquitin-mediated proteolysis pathways in ESCC. Therefore, we hypothesized that LINC00022 negatively regulates the protein level of p21 through the ubiquitin-proteasome pathway (UPP). Protein stability assay depicted that ectopic expression of LINC00022 shortened the half-life of p21 protein in the presence of CHX (Fig. 5I), an inhibitor of protein synthesis. The decrease in p21 protein levels caused by over-expression of LINC00022 was partially abrogated by the proteasome inhibitor MG132 (Fig. 5J). Moreover, co-IP combined with Western blot revealed that up-regulation of LINC00022 induced the ubiquitination of p21 protein (Fig. 5K). Further, we examined the protein expression and ubiquitination level of p21 in xenografts tumors derived from our in vivo experiment (Fig. 3C), and found that the level of p21 protein decreased and its ubiquitination increased in the OE-022 group compared with the OE-NC group (Supplementary Fig. 8B), consistent with the results of cell experiments. Collectively, these data above suggest that LINC00022 de-stabilizes p21 protein by promoting its decay via UPP.

### FTO mediates m6A demethylation of LINC00022 and promotes its expression

Both genomic and epigenetic alterations can cause the dysregulation of LncRNA expression in cancer cells [14, 43]. To explore the mechanism responsible for the up-regulation of LINC00022 in ESCC, we first analyzed the copy number variation (CNV) levels of LINC00022 gene and its promoter DNA methylation level in tumor and normal samples from the TCGA database. We showed that the genome CNV level of LINC00022 was positively correlated with its expression in ESCA samples (Supplementary Fig. 9A). In addition, tumor samples with high expression of LINC00022 had higher CNV levels (Supplementary Fig. 9B), and the LINC00022-amplified tumor samples showed higher expression of LINC00022 (Supplementary Fig. 9C). However, there was no significant correlation between DNA methylation in LINC00022 promoter and its transcription level in samples from TCGA-ESCA (Supplementary Fig. 9D-F). These data suggest that the increase of DNA copy number may be one of the genetic mechanisms for the up-regulation of LINC00022 expression.

**Fig. 6:**
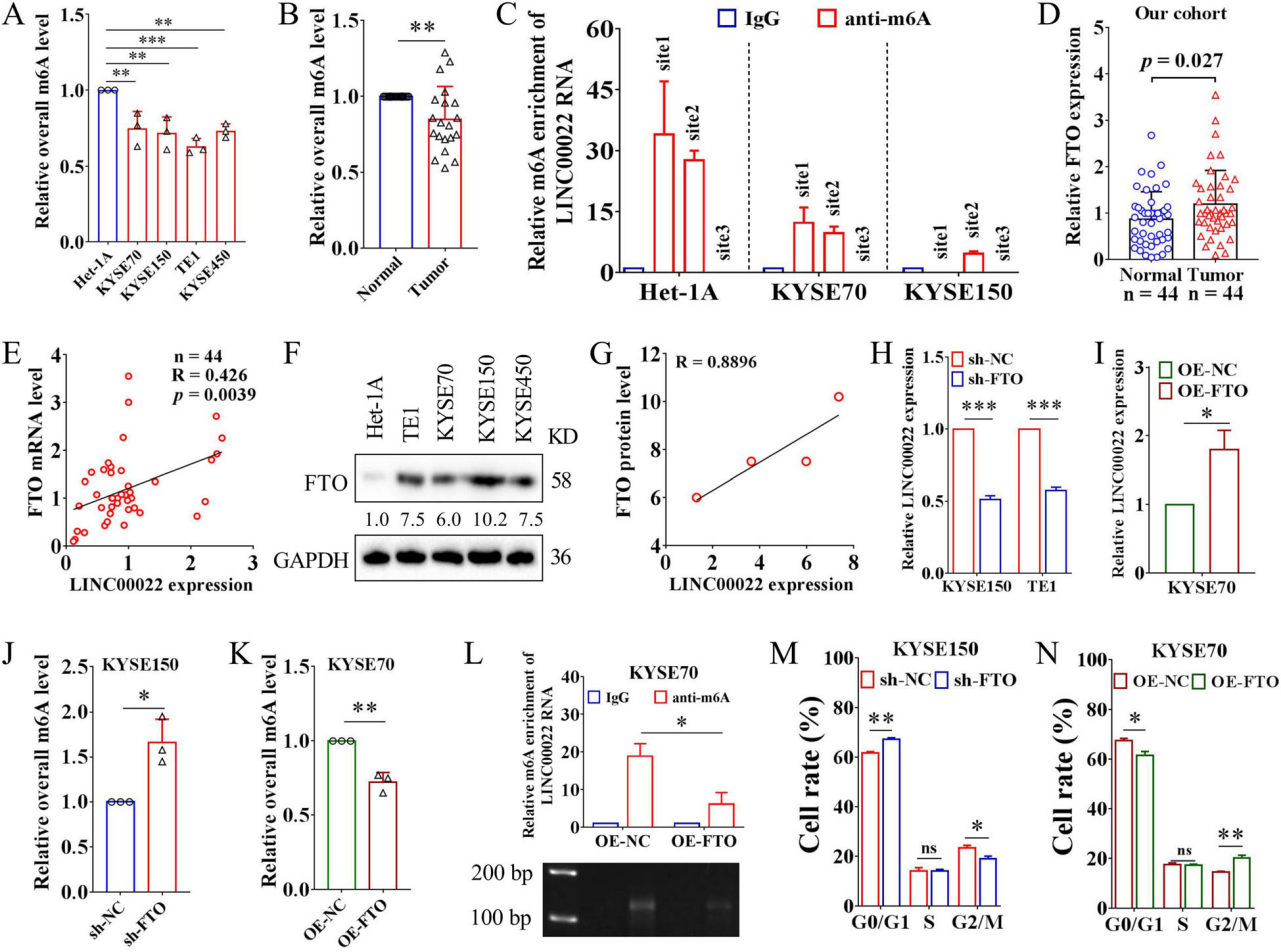
FTO mediates m6A demethylation of LINC00022 and promotes its up-regulation. (A) The colorimetric m6A detection assay showed that the overall m6A levels in ESCC cells were decreased compared with Het-1A, **p < 0.01; ***p < 0.001. (B) The relative overall m6A level in 20 pairs of ESCC tissues was tested by the colorimetric m6A detection kit, **p < 0.01. (C) MeRIP combined with specific qRT-PCR was utilized to detect the relative m6A enrichment at three sites of LINC00022 transcript in Het-1A, KYSE70 and KYSE150 cells. Three putative m6A modification sites (site1, site2, site3) of LINC00022 RNA analyzed by SRAMP and RMBase V2.0 were shown in Supplementary Fig. 10B. (D) The mRNA levels of FTO in 44 pairs of ESCC tumor tissues and adjacent normal tissues were determined by qRT-PCR. (E) Person’s Coefficient analysis showed the positive correlation between LINC00022 transcription and FTO mRNA in 44 cases of ESCC tumors. (F) The protein levels of FTO in Het-1A, TE1, KYSE70, KYSE150 and KYSE450 cell lines were examined by Western blot. (G) Person’s Coefficient analysis revealed the positive correlation between LINC00022 transcription and FTO protein in TE1, KYSE70, KYSE150 and KYSE450 cells. (H-I) Knockdown or over-expression of FTO resulted in a significant decrease or increase in LINC00022 expression in ESCC cells revealed by qRT-PCR, *p < 0.05; ***p < 0.001. (J-K) Knockdown or over-expression of FTO led to an increase or decrease in overall m6A levels of RNAs in ESCC cells analyzed by colorimetric m6A detection kit, *p < 0.05; **p < 0.01. (L) MeRIP combined with specific qRT-PCR uncovered that FTO over-expression led to a dramatic decrease in m6A enrichment (site2) of LINC00022 transcript in KYSE70 cells (upper panel, **p < 0.05). The qRT-PCR products were then examined by agarose gel electrophoresis (nether panel). (M-N) Cell cycle phase distribution of KYSE150 or KYSE70 following lentivirus-mediated knockdown or over-expression of FTO was detected by PI-staining flow cytometry, *p < 0.05; **p < 0.01; ns means no significance

Recent advances in epigenetic studies have revealed the pivotal role of m6A modification at the post-transcriptional level in RNA metabolism and function in tumor cells [44, 45]. We wondered whether m6A modification was related to the up-regulation of LINC00022 in ESCC. For the first time, we examined the overall m6A levels of RNAs in ESCC using the colorimetric m6A detection assay. Both ESCC cell lines and tumor tissues showed lower levels of RNA m6A compared to the counterparts (Fig. 6A-B), implying that m6A may be involved in the pathogenesis of ESCC. Two m6A online analysis software, SRAMP and RMBase V2.0, predicted 11 and 8 highly reliable m6A modification sites on the LINC00022 transcript (Supplementary Fig. 10A), respectively, among which 3 overlapped loci with high confidence were located at 1074 bp (site1), 1374 bp (site2) and 1462 bp (site3) from the 5′-end (Supplementary Fig. 10B). Subsequently, MeRIP combined with qRT-PCR analysis uncovered that the transcripts of LINC00022 in cell lines Het-1A, KYSE70 and KYSE150 were enriched with m6A at different levels. The m6A modification of site1 was detected in Het-1A and KYSE70 cells, while the m6A modification of site3 was not observed in Het-1A, KYSE70 and KYSE150 cells. Importantly, the m6A modification of site2 was detected in all these three cell lines. The enrichment level of m6A was the highest in Het-1A and the lowest in KYSE150 (Fig. 6C), which is contrary to LINC00022 expression in these three ESCC cell lines, suggesting that m6A modification may play an important role in the regulation of LINC00022.

To further define which m6A enzyme is involved in the regulation of LINC00022, we conducted bioinformatics analysis using the online tool m6A2Target based on sequencing validation data. We found that METTL3, KIAA1429 and FTO may interfere with the expression of LINC00022 (Supplementary Fig. 11A). Further analysis using GEPIA showed that among the three m6A regulators, only FTO was highly expressed in ESCC and was positively correlated with LINC00022 in 182 tumor samples (Supplementary Fig. 11B-D). Therefore, we focused on the role of FTO in ESCC and its regulatory effect on LINC00022. We then analyzed the mRNA expression of FTO in our in-house cohort containing 44 pairs of ESCC specimens by qRT-PCR. FTO was up-regulated in ESCC tumors and positively correlated with LINC00022 expression (Fig. 6D-E). Moreover, the elevation of FTO and its positive correlation with the expression of LINC00022 were also found in a panel of ESCC cell lines (Fig. 6F-G). We used a Lentivirus-mediated system to specifically reduce or increase the intracellular level of FTO in ESCC cells and verified the efficiencies of FTO manipulation by qRT-PCR and Western blot (Supplementary Fig. 12A-B). In KYSE150 and TE1 cells, FTO knockdown resulted in a significant down-regulation of LINC00022 expression (Fig. 6H). In KYSE70 cells, LINC00022 expression was evidently increased after FTO over-expression (Fig. 6I). Neither deletion nor up-regulation of LINC00022 significantly affected the expression level of FTO in ESCC cells (Supplementary Fig. 12C-D), suggesting that FTO is an upstream molecule of LINC00022.

The colorimetric m6A detection assay revealed that knockdown or over-expression of FTO resulted in the increase or decrease of the overall m6A modification level in ESCC cells (Fig. 6J-K), respectively, which further indicated the key role of FTO in the epigenetic regulation of ESCC. Considering that FTO positively regulates the expression of LINC00022, MeRIP combined with qRT-PCR was performed to investigate whether FTO mediated the m6A modification of LINC00022 transcript at site2 in ESCC cells. The results showed that ectopic expression of FTO in KYSE70 cells significantly reduced the enrichment of m6A at site2 of the LINC00022 transcript (Fig. 6L). Both loss-of-function and gain-of-function experiments demonstrated that FTO was an oncogenic factor to facilitate proliferation (Supplementary Fig. 12E-F) and cell-cycle progression (Fig. 6M-N, Supplementary Fig. 12G-H) in ESCC cells, similar to the function of LINC00022. Clinically, survival analysis from TCGA-ESCC cohort depicted those patients with high FTO expression had a shorter median survival than those with low FTO expression (Supplementary Fig. 13A, HR = 1.43, *p* = 0.38, 25.4 months vs. 42.1 months). We also analyzed the relationship between FTO and clinical parameters of 36 patients with complete information in our in-house cohort. FTO was up-regulated in tumor tissues of female patients or advanced stage patients, compared with male or early-stage patients (Supplementary Fig. 13B), respectively. No significant correlation was found between FTO expression and age, differentiation, grade, lymph node metastasis or depth of invasion (Supplementary Fig. 13C). Taken together, these data suggest that up-regulated FTO mediates the RNA m6A demethylation of LINC00022 in ESCC cells and promotes its expression, thus, enhancing ESCC cell proliferation.

### FTO promotes LINC00022 up-regulation in an YTHDF2-dependent manner

**Fig. 7:**
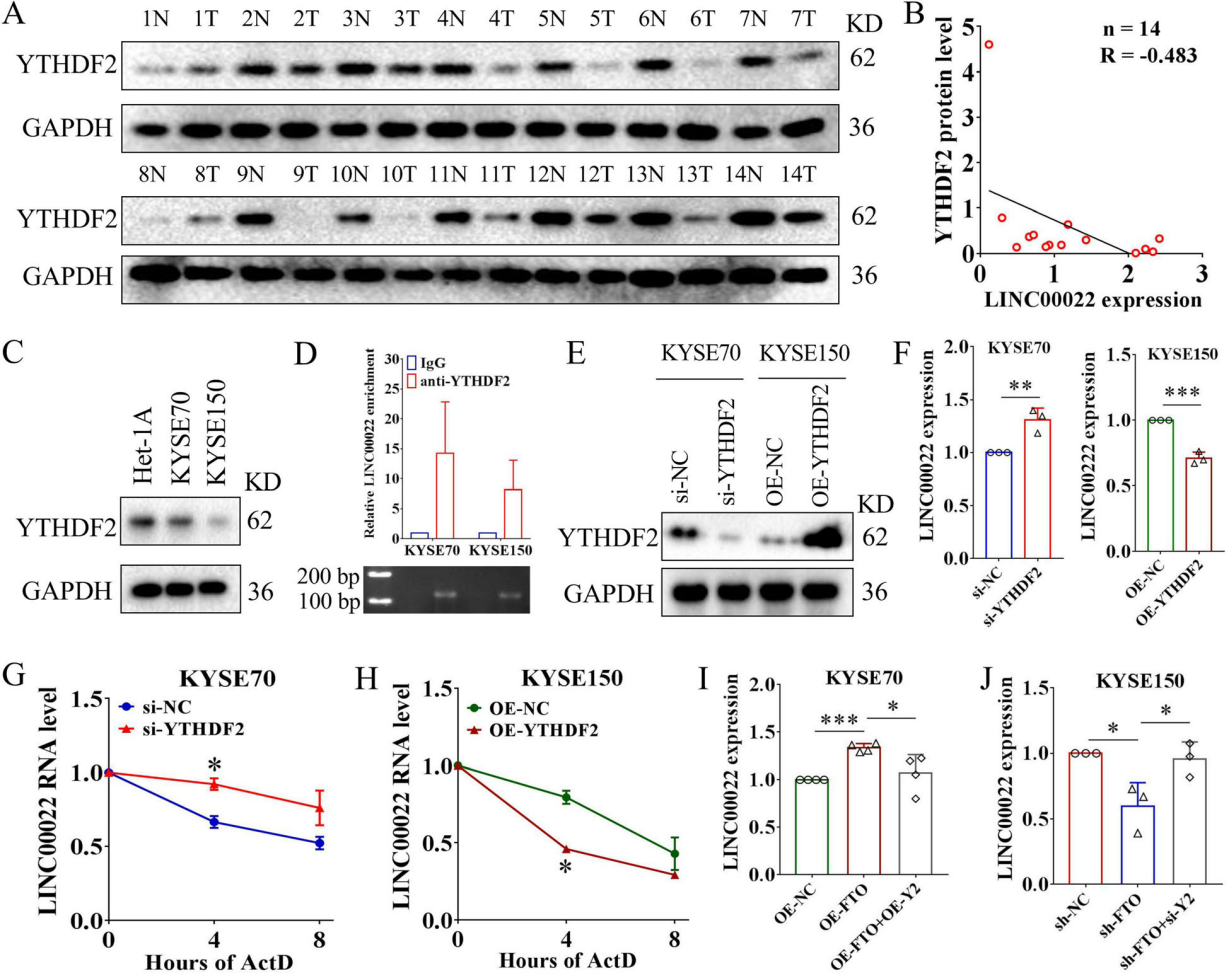
FTO-induced epigenetic up-regulation of LINC00022 is YTHDF2-dependent. (A) Western blot was used to examine the protein levels of YTHDF2 in 14 pairs of ESCC tissues. N represents normal and T represents tumor. (B) Person’s Coefficient analysis revealed the negative correlation between YTHDF2 protein and LINC00022 transcription in 14 cases of ESCC tumors. (C) The protein levels of YTHDF2 in Het-1A, KYSE70 and KYSE150 was examined by Western blot. (D) The direct binding affinity of LINC00022 transcript with YTHDF2 protein was determined by RIP-qRT-PCR (upper panel) and agarose gel electrophoresis (nether panel). (E) The knockdown or over-expression efficiencies of YTHDF2 mediated by recombinant lentivirus were verified by Western blot in KYSE70 and KYSE150 cells. (F) Knockdown or over-expression of YTHDF2 resulted in a significant increase or decrease in LINC00022 expression in ESCC cells revealed by qRT-PCR, **p < 0.01; ***p < 0.001. (G-H) The effect of knockdown or over-expression of YTHDF2 on the stability of LINC00022 transcript in ESCC cells in the presence of ActD (5 μg/mL) was tested by qRT-PCR, *p < 0.05. (I) Knockdown of YTHDF2 (si-Y2) significantly abolished FTO ablation-induced down-regulation of LINC00022 in KYSE150 cells, *p < 0.05. (J) The up-regulation of LINC00022 caused by FTO over-expression was relieved by increase of YTHDF2 (OE-Y2) in KYSE70 cells, *p < 0.05; ***p < 0.001

The m6A readers play crucial roles in determining the fate of m6A-marked RNAs, such as stabilization and degradation [46]. It has been found that, unlike other m6A readers that stabilize RNA, YTHDF2 tends to de-stabilize m6A-modified RNA molecules and facilitates their degradation [47, 48]. Our above results showed that the expression level of LINC00022 was increased in ESCC cells (Fig. 1F), while its m6A modification level was decreased (Fig. 6C). We therefore hypothesized that YTHDF2 might regulate the m6A-modified LINC00022 transcript in ESCC cells. To confirm this hypothesis, we first determined the protein levels of YTHDF2 in 14 pairs of ESCC tumor specimens and the matched normal specimens by Western blot, and found that YTHDF2 was decreased in 13 of the 14 cases of ESCC tumors (Fig. 7A). Pearson’s coefficient analysis showed the inverse correlation between YTHDF2 and LINC00022 expression in 14 cases of ESCC tumors (Fig. 7B). Lower YTHDF2 levels were also detected in KYSE70 and KYSE150 cell lines, compared with Het-1A (Fig. 7C). RIP analysis confirmed the direct binding between YTHDF2 protein and LINC00022 transcript in KYSE70 and KYSE150 cells (Fig. 7D). We used siRNAs to down-regulate the intracellular level of YTHDF2 and a plasmid-mediated system to increase the expression of YTHDF2 in ESCC cells (Fig. 7E). The level of LINC00022 in KYSE70 cells was significantly up-regulated, albeit modestly, after YTHDF2 deletion (Fig. 7F). Ectopic expression of YTHDF2 led to an obvious decrease in LINC00022 level in KYSE150 cells (Fig. 7F). Importantly, ablation of YTHDF2 slowed down the decay rate of LINC00022 in ESCC cells, while over-expression of YTHDF2 accelerated the degradation of LINC00022 transcript in the presence of Act D (Fig. 7G-H), an RNA synthesis inhibitor. We next investigated the biological function of YTHDF2 in ESCC, and found that over-expression of YTHDF2 inhibited the proliferation and cell cycle progression of ESCC cells (Supplementary Fig. 14A-B), while knockdown of YTHDF2 had the opposite effect (Supplementary Fig. 14C-D). Clinically, Kaplan-Meier analysis of TCGA-ESCC dataset showed that high level of YTHDF2 was a favorable factor for patient OS (Supplementary Fig. 14E). This indicates that, contrary to LINC00022 and FTO, YTHDF2 plays a tumor suppressor role in ESCC. Furthermore, we confirmed that YTHDF2 deletion rescued the decrease in LINC00022 levels caused by FTO knockdown in KYSE150 cells (Fig. 7I). Enforced YTHDF2 abolished the up-regulation of LINC00022 induced by ectopic FTO expression in KYSE70 cells (Fig. 7J). These findings indicate that FTO promotes LINC00022 up-regulation in ESCC cells via an m6A-YTHDF2-dependent mechanism.

### The FTO/LINC00022 axis drives ESCC in vitro and in vivo

**Fig. 8:**
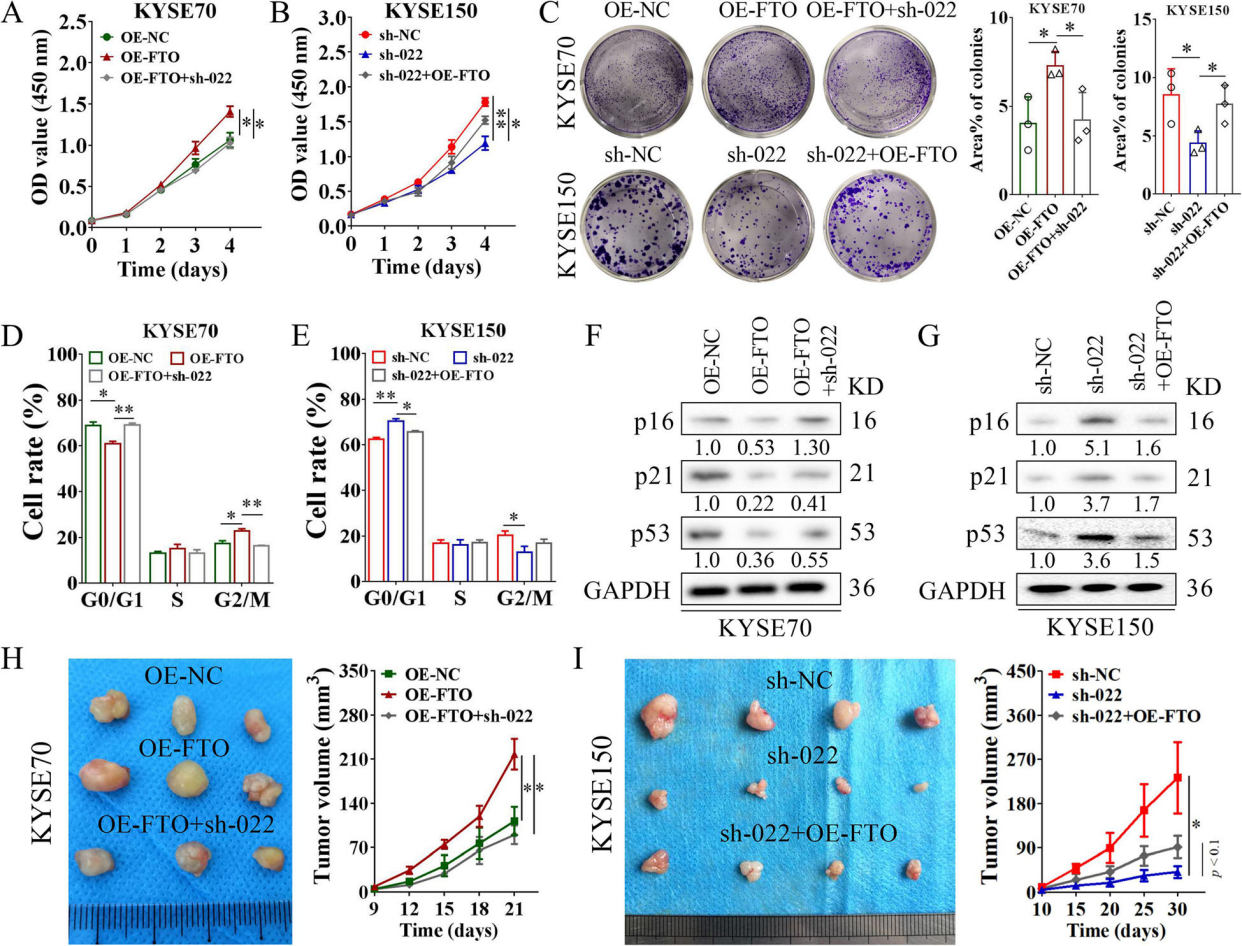
FTO/LINC00022 axis drives ESCC tumorigenesis. (A) Deletion of LINC00022 attenuated the proliferative effects induced by FTO over-expression in KYSE70 cells, *p < 0.05. (B) Ectopic expression of FTO partially relieved the inhibitory effects of proliferation caused by LINC00022 ablation in KYSE150 cells, *p < 0.05; **p < 0.01. (C) The non-population-dependent growth ability of ESCC cells under the control of the FTO/LINC00022 axis was evaluated by the colony formation assay, *p < 0.05. (D-E) PI-labeling staining combined with flow cytometry indicated the role of FTO/LINC00022 axis in ESCC cell-cycle progression, *p < 0.05; **p < 0.01. (F-G) Western blot was used to investigate the changes of cell cycle regulator protein levels, p16, p21 and p53 in KYSE70 (F) and KYSE150 (G) cells following FTO-LINC00022 cross-talking. (H) Knockdown of LINC00022 fully rescued the growth promotion effect induced by ectopic FTO expression on KYSE70 cells in nude mice (*n* = 3) (left panel). The tumor volume was measured every three days from day 9 to day 21 after cell inoculation, and the curve was plotted (right panel), *p < 0.05. (I) Over-expression of FTO partially attenuated the inhibition of subcutaneous tumorigenicity caused by LINC00022 knockdown on KYSE150 cells in nude mice (*n* = 4) (left panel). The tumor volume was monitored every five days from day 10 to day 30 after cell inoculation, and the curve was generated (right panel), *p < 0.05

To elucidate the role of the FTO/LINC00022 axis in tumorigenesis of ESCC, we carried out functional rescue experiments in vitro and in vivo. CCK-8 detection showed that knockdown of LINC00022 almost fully abolished the pro-proliferative effect of FTO on ESCC cells (Fig. 8A). Genetic addition of FTO effectively relieved the inhibitory impact of LINC00022 knockdown on the proliferation of ESCC cells (Fig. 8B). The regulation of FTO/LINC00022 cross-talk on ESCC cell growth was further confirmed by colony formation assays (Fig. 8C). PI-labeling flow cytometry revealed that LINC00022 deletion significantly retarded FTO-induced cell-cycle progression in KYSE70 cells (Fig. 8D, Supplementary Fig. 15A) while over-expression of FTO partially alleviated the G0/G1 phase arrest elicited by ablation of LINC00022 in KYSE150 cells (Fig. 8E, Supplementary Fig. 15B). Concomitantly, over-expression of FTO reduced the protein levels of p16, p21, and p53 in KYSE70 cells, and knockdown of LINC00022 counteracted, at least in part, these effects (Fig. 8F). Moreover, ectopic expression of FTO blocked the elevation of p16, p21, and p53 caused by LINC00022 deficiency in KYSE150 cells (Fig. 8G). The xenografts experiment in nude mice further demonstrated that ablation of LINC00022 completely mitigated FTO-induced cell proliferation of KYSE70 in vivo (Fig. 8H, Supplementary Fig. 15C), whereas over-expression of FTO partially relieved the inhibitory effect of tumor formation caused by LINC00022 knockdown (Fig. 8I, Supplementary Fig. 15D). Collectively, these data indicate that the FTO/LINC00022 axis regulates ESCC growth in vitro and in vivo.

## Discussion

In the past few decades, most studies in molecular oncology focused on exploring the function of protein-coding genes. As the recent advances in high-throughput sequencing technology, LncRNAs, formerly known as the “dark matter” of the genome, have been found to be widely expressed in mammalian cells and to be clearly involved in human diseases [49, 50]. Thus, LncRNAs have been considered as promising biomarkers and targets for the diagnosis and treatment of cancer due to their tissue-specific characteristics and important roles in reshaping tumor cell growth and survival [23, 51]. However, the biological role of LncRNA in ESCC development and the upstream mechanism controlling the dysregulation of LncRNA remain largely unclear. Here we show the critical role of m6A-mediated epigenetic up-regulation of LINC00022 in ESCC tumorigenesis.

Through sequencing and microarray techniques, a considerable number of abnormally expressed LncRNAs were discovered in ESCC tumor samples compared with adjacent normal samples [14, 52]. Indeed, identification of ESCC-related subsets among the thousands of LncRNAs remains a challenge. Although several functionally described LncRNAs, such as ESCCAL-1 [14] and CCAT1 [28], have been linked to the malignant behaviors of ESCC, the specific roles and precise mechanisms of most LncRNAs in ESCC biology have not been characterized. This study identified a novel LncRNA named LINC00022 that was significantly up-regulated in ESCC tumors by interrogating the array-based dataset GSE75241 and the sequencing-based dataset TCGA-ESCC. Furthermore, the over-expressed status of LINC00022 in ESCC was verified by three additional datasets, including our own in-house cohort. There has been no specific biomarker for the early diagnosis and outcome prediction of ESCC clinically, which is the bottleneck for improving the prognosis in patients with ESCC [12, 53]. Exploring aberrant regulation of LncRNAs has brought a new avenue to address the mechanism of oncogenic regulator and potential therapeutic strategy for human cancers including ESCC [54–56]. A recent study reported that LncRNA detection based on plasma extracellular vesicles has a high diagnostic value for pancreatic ductal carcinoma [54]. In this work, we for the first time comprehensively analyzed the expression profile and prognostic correlation of LINC00022 in human pan-cancer across 29 cancer types, including ESCC, based on GEPIA database. Surprisingly, we found that LINC00022 showed consistent expression status and prognostic significance only in ESCC and not in other types of cancer; LINC00022 was highly expressed in ESCC tumor samples, and patients with high expression of LINC00022 had a shorter survival. Importantly, LINC00022 deletion significantly suppressed the growth of ESCC cells in vitro and the tumorigenicity in vivo. These findings together suggest that LINC00022 is an ESCC-specific LncRNA, providing a scientific basis for targeting LINC00022 in the treatment of ESCC. Considering the clinical perspective of LINC00022 as early diagnosis and prognosis marker in ESCC, a large number of samples and especially blood or humoral origins are needed to be further investigated and validated.

Mechanistically, continuous activation of cell cycle signal is one of the predominant biological characteristics of malignant tumor progression, and anti-tumor drugs based on targeting cell-cycle regulators, such as CDK inhibitors, have been proved to be effective [42, 57]. However, inherent or acquired resistance of cancer cells to CDK inhibitors has become a major obstacle to their clinical application [58, 59]. Alternative strategies to modulate cell cycle regulators in cancer cells are therefore anticipated. Here, we demonstrated that LINC00022 promoted cell cycle progression of ESCC cells by affecting a series of cell cycle regulators including p16, p21, p53, CDK2 and Cyclin E1. More precisely, LINC00022 promotes instability of p21 protein in ESCC cells via UPP. Although we have yet to clarify the pathways involved in LINC00022-mediated changes in other cell cycle regulators, our study reveals a novel mechanism of LncRNA-mediated post-translational modification of p21 protein in ESCC cells. To our knowledge, this is the first report that p21 protein can bind to and be regulated by LncRNA, providing novel insights into targeting p21 against ESCC through LINC00022.

In contrast to epigenetic modifications that regulate gene expression at the transcriptional level, such as DNA methylation and histone acetylation, the dissecting mechanism of m6A modification on RNA has greatly improved the understanding of gene regulation at the post-transcriptional level in eukaryotic cells [60]. Dynamic m6A modification of mRNA has been shown to be necessary for retaining cell growth, metabolism and differentiation [61, 62]. Tumor cells maintain their self-renewal and proliferation under different conditions by orchestrating abnormal m6A modifications of specific mRNAs [63–65]. A large number of recent studies have focused on exploring the role of m6A-methylated mRNA in cancer cells, and some LncRNAs such as LINC00958 [66], DANCR [67], and MALAT1 [68] modified by m6A have also been reported. But the regulation of LncRNA by m6A modification in ESCC is still poorly understood. In our study, bioinformatics analysis and MeRIP assay together revealed that the m6A-modified LINC00022 transcript presents in ESCC cells. Furthermore, the LINC00022 transcript in ESCC cells showed a lower level of m6A modification than that of normal cell line Het-1A, which was regulated by the m6A demethylase FTO. To our knowledge, this is the first report that the stability of LncRNA in ESCC cells can be regulated by m6A, expanding our understanding of LncRNA participating in the pathogenesis of ESCC.

The function of m6A-labeled RNA in cells is mainly determined by the m6A-reading protein [46, 48]. Unlike other m6A readers, such as IGF2BPs, YTHDCs, HNRNPs, YTHDF1 and YTHDF3, which have been shown to promote RNA stabilization, splicing or translation, YTHDF2 appears to be more inclined to accelerate the degradation of m6A-marked RNA [44, 46, 47]. YTHDF2 mediates the attenuation of oncogenic mRNAs and participates in the inhibition of colorectal cancer [69] or enhances the anti-tumor effect of natural killer cells [70]. However, we have not seen any reports on the function of YTHDF2 in ESCC. In this study, we showed that the protein levels of YTHDF2 in ESCC tissues and cells were significantly declined compared with the counterparts, and YTHDF2 protein could bind to the LINC00022 transcript and promote its decay in ESCC cells. Over-expression of YTHDF2 inhibited proliferation and cell cycle progress in ESCC cells, indicating its tumor suppressive role in ESCC. Different from our findings and other studies [69, 70], a recent study reported that YTHDF2 maintains the viability of glioblastoma stem cells by stabilizing MYC mRNA [71]. These results suggest that YTHDF2, as an m6A reader, is more complicated in the regulation of different tumors or cell-context dependent than we currently realized.

FTO was first identified to be a tumor-promoting factor in acute myeloid leukemia as an m6A demethylase [72]. FTO has since been widely reported to function as an oncogene in a variety of cancer types. FTO mediates the m6A demethylation of mRNA or LncRNA, and regulates the stability of the m6A-modified RNA by cooperating with the m6A reader [73–75]. In this work, our data suggested that LINC00022 is a novel downstream target of FTO, and FTO mimics the promoting effect of oncogenic LINC00022 on ESCC tumorigenesis. We also observed that in functional rescue experiments, deletion of LINC00022 almost completely alleviated the proliferation and tumor growth of ESCC induced by over-expression of FTO, while enforced FTO partially relieved the growth inhibition of ESCC cells mediated by LINC00022 deficiency in vitro and in vivo. This suggests that FTO may not be the only upstream regulator of LINC00022 in ESCC cells, which needs to be further explored. Additionally, by analyzing the TCGA-ESCA dataset, we found a high copy number of LINC00022 gene in tumor samples, implying that gene amplification and m6A modification may work together mediating the up-regulation of LINC00022.

## Conclusions

In conclusion, our findings reveal the driving role of LINC00022 and FTO in ESCC tumorigenesis. LINC00022 interacts with p21 protein and facilitates its instability via UPP. The m6A demethylase FTO mediates epigenetic up-regulation of LINC00022 in ESCC cells in an YTHDF2-dependent manner. The FTO/LINC00022 axis promotes ESCC growth both in vitro and in vivo, providing a proof of concept for the novel therapeutic vulnerability of ESCC.

## Supplementary Information

**Additional file 1: Suppl. Fig. 1** LINC00022 is up-regulated in EAC cohort from TCGA program. (A) The paired result of LINC00022 expression in 44 cases of ESCC was shown. (B) The elevation of LINC00022 was found in EAC tumors from TCGA cohort (11 normal tissues vs. 80 tumor tissues). (C) When both ESCC and EAC samples from TCGA were included, the expression of LINC00022 was also higher in tumors than that in normal tissues (11 normal tissues vs. 81 ESCC tumor tissues + 80 EAC tumor tissues).

**Additional file 2: Suppl. Fig. 2** Pan-cancer analysis based on GEPIA database reveals the expression landscape of LINC00022 in human cancers. The comprehensive cancer database GEPIA was employed to investigate the expression landscape of LINC00022 in 28 types of cancer. (A) LINC00022 was significantly increased in cervical squamous carcinoma (CESC, 13 N vs. 306 T), lymphoid neoplasm diffused large B-cell lymphoma (DLBC, 337 N vs. 47 T), glioblastoma (GBM, 207 N vs. 163 T), skin cutaneous melanoma (SKCM, 558 N vs. 461 T), stomach adenocarcinoma (STAD, 211 N vs. 408 T) and thymoma (THYM, 339 N vs. 118 T), while decreased in testicular germ cell tumor (TGCT, 165 N vs. 137 T). (B-D) LINC00022 expression showed no significant differences between tumor samples and normal samples in adrenocortical carcinoma (ACC, 128 N vs. 77 T), bladder urothelial carcinoma (BLCA, 28 N vs. 404 T), breast cancer (BRCA, 291 N vs. 1085 T), cholangio carcinoma (CHOL, 9 N vs. 36 T), colon adenocarcinoma (COAD, 349 N vs. 275 T), head and neck squamous cell carcinoma (HNSC, 44 N vs. 519 T), kidney chromophobe (KICH, 53 N vs. 66 T), kidney renal clear cell carcinoma (KIRC, 100 N vs. 523 T), kidney renal papillary cell carcinoma (KIRP, 607 N vs. 286 T), acute myeloid leukemia (LAML, 707 N vs. 173 T), low grade glioma (LGG, 207 N vs. 518 T), liver hepatocellular carcinoma (LIHC, 160 N vs. 369 T), lung adenocarcinoma (LUAD, 347 N vs. 483 T), lung squamous cell carcinoma (LUSC, 338 N vs. 486 T), ovarian carcinoma (OV, 88 N vs. 426 T), pancreatic adenocarcinoma (PAAD, 171 N vs. 179 T), prostate adenocarcinoma (PRAD, 1521 N vs. 492 T), rectum adenocarcinoma (READ, 318 N vs. 92 T), thyroid carcinoma (THCA, 337 N vs. 512 T), uterine corpus rndometrial carcinoma (UCEC, 91 N vs. 174 T) and uterine carcinosarcoma (USC, 78 N vs. 57 T).

**Additional file 3: Suppl. Fig. 3** Prognostic analysis of LINC00022 in CESC, DLBC, GBM, SKCM, STAD, TGCT and THYM based on GEPIA database. GEPIA was utilized to analyze the prognostic significance of LINC00022 in seven types of cancer with dysregulated expression. LINC00022 expression was not significantly associated with OS in patients with CESC, DLBC, GBM, SKCM, STAD and TGCT, except for THYM.

**Additional file 4: Suppl. Fig. 4** Relationships between LINC00022 expression and clinical characteristics of ESCC patients in our study cohort. (A) The expression of LINC00022 in tumor tissues of female patients was obviously higher than that of male patients, *p* = 0.046. (B) LINC00022 expression in tumor tissues of stage III-IV patients was significantly higher than that in tumor tissues of stage I-II patients, *p* = 0.044. (B-C) No significant correlation was found between the expression of LINC00022 and age, differentiation, grade, lymph node metastasis or depth of invasion.

**Additional file 5: Suppl. Fig. 5** The diagnostic and prognostic values of LINC00022 in various ESCC cohorts. (A) ROC analysis suggested the high diagnostic value of LINC00022 in the TCGA-ESCC and TCGA-EAC cohorts (AUC > 0.9 and *p* < 0.0001). (B) The AUC value of LINC00022 in CRN cohort was as high as 0.9795 (*p* < 0.0001). (C) The prognostic significance of LINC00022 in GEPIA cohort was determined by the Kaplan-Meier method. Elevated LINC00022 expression indicated worse patient DFS. (D-E) Kaplan-Meier analysis from TCGA-ESCC and TCGA-EAC cohorts showed that patients with higher LINC00022 expression had a shorter median OS.

**Additional file 6: Suppl. Fig. 6** KEGG pathway analysis reveals the crucial role of LINC00022 in ESCC. 1000 genes associated with LINC00022 in ESCC tumors was obtained from GEPIA database and subjected to KEGG pathway analysis. The enrichment result was shown as a bubble chart. KEGG analysis depicted that LINC00022 may be mainly involved in mismatch repair, DNA replication, cell cycle, spliceosome, nucleotide excision repair, mRNA surveillance pathway, proteasome, pyrimidine metabolism, ubiquitin mediated proteolysis, and purine metabolism.

**Additional file 7: Suppl. Fig. 7** Knockdown of LINC00022 has no significant impact on apoptosis of ESCC cells. (A) Annexin V-FITC/PI double staining and flow cytometry was used to detect apoptosis of KYSE150 and TE1 cells following LINC00022 silence. (B) PI-labeling combined with fluorescent microscope revealed that LINC00022 did not contribute to cell apoptosis of ESCC.

**Additional file 8: Suppl. Fig. 8** The protein expression and ubiquitination level of p21 in LINC00022-augmented tumors. (A) The prognostic significance of p21 in TCGA-ESCC cohort was analyzed by the Kaplan-Meier method. Elevated p21 expression indicated better patient DFS. (B) Co-IP and Western blot were performed to examine the protein level and ubiquitination of p21 in OE-NC and OE-022 tumors derived from the in vivo experiment.

**Additional file 9: Suppl. Fig. 9** Copy number variation (CNV), rather than DNA methylation, correlates with LINC00022 expression in ESCA. (A) The genome CNV level of LINC00022 was positively correlated with its expression in 159 cases of ESCA samples from TCGA, Cor = 0.35 and *p* = 1.3E-5. (B) Tumor samples with high expression of LINC00022 had higher CNV level. The 159 cases of tumor samples were divided into High and Low groups according to the upper 95 quantile of LINC00022 expression in normal samples as the threshold, *p* = 0.0015. (C) The gene amplified tumor samples showed higher expression of LINC00022. The 159 cases of tumor samples were divided into Gain and Normal/Loss groups according to logratio value 0 of copy number as the threshold. (D) The methylation level of LINC00022 promoter showed no significant differences between tumor and normal samples in ESCA. (E-F) The methylation level of LINC00022 promoter was not significantly associated with its expression in ESCA samples from TCGA.

**Additional file 10: Suppl. Fig. 10** LINC00022 transcript contains many potential m6A modification sites. (A) The algorithm of software SRAMP and RMBase V2.0 analyzed 11 and 8 highly reliable m6A sites on the LINC00022 transcript, respectively. (B) Three overlapped m6A loci with high confidence were located at 1074 bp, 1374 bp, 1462 bp from 5′-end on the LINC00022 transcript.

**Additional file 11: Suppl. Fig. 11** FTO is up-regulated in ESCC and positively correlated with LINC00022 expression. (A) The online tool m6A2Target, based on sequencing validation data, was used to analyze m6A modification enzymes that may perturb LINC00022 expression. (B-D) The expression of FTO, METTL3 and KIAA1429 in ESCC and their correlation with LINC00022 was analyzed by the GEPIA database.

**Additional file 12: Suppl. Fig. 12** FTO promotes ESCC proliferation and cell-cycle progression. (A) The knockdown efficiencies of FTO mediated by recombinant lentivirus were validated at the mRNA and protein levels in KYSE150 and TE1 cells by qRT-PCR (upper panel, **p < 0.01; ***p < 0.001) and Western blot (nether panel), respectively. (B) The over-expression efficiencies of FTO mediated by recombinant lentivirus were validated at the mRNA and protein levels in KYSE70 cells by qRT-PCR (upper panel, **p < 0.01) and Western blot (nether panel), respectively. (C-D) Both the ablation and over-expression of LINC00022 had no significant effect on the expression on FTO in ESCC cells as depicted by qRT-PCR. (E-F) CCK-8 assay was employed to evaluate cell viability of KYSE150 with FTO knockdown (A), and KYSE70 with FTO over-expression (B), *p < 0.05. (G-H) Cell cycle phase distribution of KYSE150 or KYSE70 following FTO knockdown or over-expression was detected by PI-staining flow cytometry.

**Additional file 13: Suppl. Fig. 13** Relationships between FTO expression and clinical characteristics of ESCC patients in our study cohort. (A) Kaplan-Meier analysis from TCGA-ESCC cohort showed that patients with higher FTO expression had a shorter median OS. (B) The expression of FTO in tumor tissues of female patients was obviously higher than that of male patients, *p* = 0.033. FTO expression in tumor tissues of stage III-IV patients was significantly higher than that in tumor tissues of stage I-II patients, *p* = 0.0039. (C) No significant correlation was found between the expression of FTO and age, differentiation, grade, lymph node metastasis or depth of invasion.

**Additional file 14: Suppl. Fig. 14** YTHDF2 suppresses ESCC proliferation and cell-cycle progression. (A) CCK-8 experiment was performed to evaluate cell proliferation of KYSE150 with YTHDF2 over-expression, **p < 0.01. (B) Flow cytometry was utilized to examine cell cycle changes of KYSE150 after over-expression of YTHDF2, **p < 0.01. (C) Knockdown of YTHDF2 enhanced cell proliferation of KYSE70 cells, *p < 0.05. (D) Silencing of YTHDF2 promoted cell cycle progression of KYSE70 cells, *p < 0.05; **p < 0.01. (E) The prognostic significance of YTHDF2 in TCGA-ESCC cohort was analyzed by the Kaplan-Meier method. Increased YTHDF2 expression indicated better patient OS.

**Additional file 15: Suppl. Fig.** 15 FTO/LINC00022 axis regulates cell-cycle and tumorigenesis of ESCC. (A-B) PI-labeling staining combined with flow cytometry revealed the role of FTO/LINC00022 axis in ESCC cell-cycle progression. (C) Knockdown of LINC00022 fully rescued the growth promotion effect caused by ectopic FTO expression on KYSE70 cells in nude mice as indicated by tumor weight (*n* = 3), *p < 0.05. (D) Over-expression of FTO partially attenuated the inhibition of subcutaneous tumorigenicity induced by LINC00022 knockdown on KYSE150 cells in nude mice as indicated by tumor weight (*n* = 4), *p < 0.05

**Additional file 16.** Primers for qRT-PCR.

## Data Availability

All data analyzed in this study are available from the corresponding author upon reasonable request.

## Abbreviations

LncRNA: Long non-coding RNA
ESCC: Esophageal squamous cell carcinoma
m6A: N6-methyladenosine
ESCA: Esophageal cancer
EAC: Esophageal adenocarcinoma
OS: Overall survival
DFS: Disease-free survival
CDKI: Cyclin-dependent kinase inhibitor
GEO: Gene Expression Omnibus
TCGA: The Cancer Genome Atlas
ROC: Receiver operating character
CNV: Copy number variation
qRT-PCR: Real-time fluorescence quantitative PCR
CCK-8: Cell counting kit 8
PI: Propidium Iodide
RIP: RNA-protein immunoprecipitation
Co-IP: Co-immunoprecipitation
MeRIP: Methylated RNA immunoprecipitation
