# Supplementary figures and images for: RNA m6A demethylase FTO-mediated epigenetic up-regulation of LINC00022 promotes tumorigenesis in esophageal squamous cell carcinoma
Source: J Exp Clin Cancer Res. 2021 Sep 20;40:294. doi: 10.1186/s13046-021-02096-1 (PMC8451109; doi:10.1186/s13046-021-02096-1)

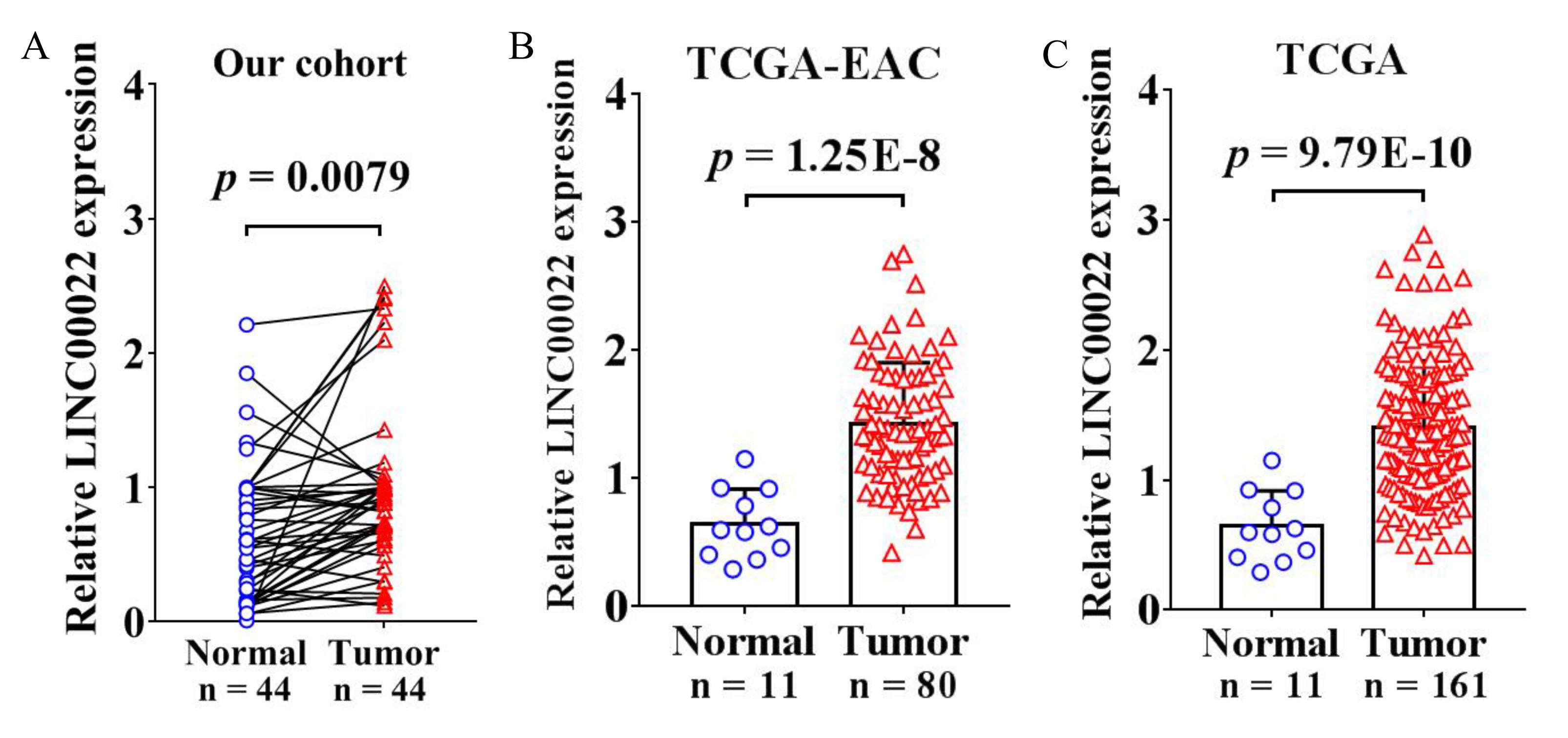

Supplement: Supplementary file 1 — Additional file 1: Suppl. Fig. 1 LINC00022 is up-regulated in EAC cohort from TCGA program. (A) The paired result of LINC00022 expression in 44 cases of ESCC was shown. (B) The elevation of LINC00022 was found in EAC tumors from TCGA cohort (11 normal tissues vs. 80 tumor tissues). (C) When both ESCC and EAC samples from TCGA were included, the expression of LINC00022 was also higher in tumors than that in normal tissues (11 normal tissues vs. 81 ESCC tumor tissues + 80 EAC tumor tissues). [file 13046_2021_2096_MOESM1_ESM.jpg]

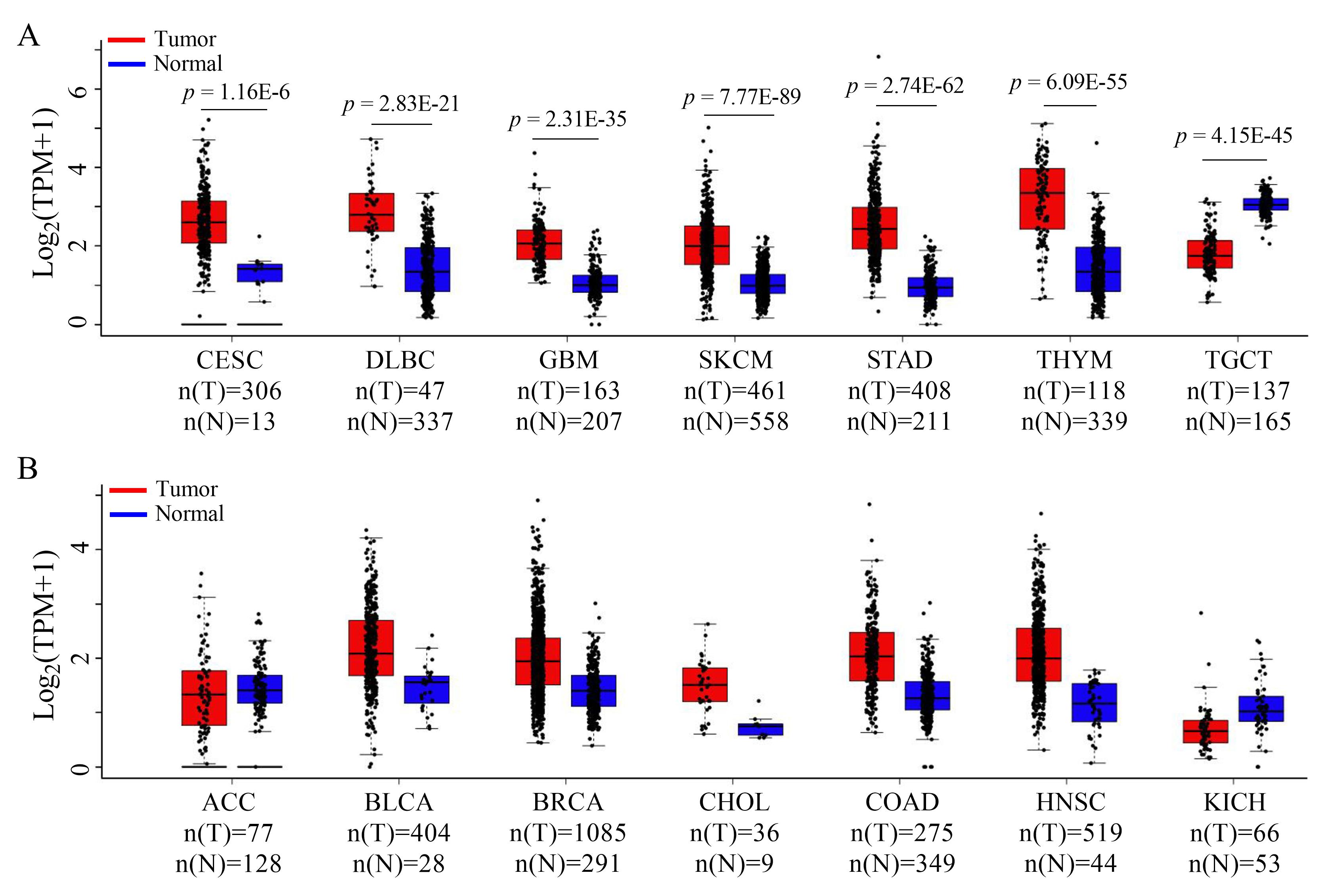

Supplement: Supplementary file 2 — Additional file 2: Suppl. Fig. 2 Pan-cancer analysis based on GEPIA database reveals the expression landscape of LINC00022 in human cancers. The comprehensive cancer database GEPIA was employed to investigate the expression landscape of LINC00022 in 28 types of cancer. (A) LINC00022 was significantly increased in cervical squamous carcinoma (CESC, 13 N vs. 306 T), lymphoid neoplasm diffused large B-cell lymphoma (DLBC, 337 N vs. 47 T), glioblastoma (GBM, 207 N vs. 163 T), skin cutaneous melanoma (SKCM, 558 N vs. 461 T), stomach adenocarcinoma (STAD, 211 N vs. 408 T) and thymoma (THYM, 339 N vs. 118 T), while decreased in testicular germ cell tumor (TGCT, 165 N vs. 137 T). (B-D) LINC00022 expression showed no significant differences between tumor samples and normal samples in adrenocortical carcinoma (ACC, 128 N vs. 77 T), bladder urothelial carcinoma (BLCA, 28 N vs. 404 T), breast cancer (BRCA, 291 N vs. 1085 T), cholangio carcinoma (CHOL, 9 N vs. 36 T), colon adenocarcinoma (COAD, 349 N vs. 275 T), head and neck squamous cell carcinoma (HNSC, 44 N vs. 519 T), kidney chromophobe (KICH, 53 N vs. 66 T), kidney renal clear cell carcinoma (KIRC, 100 N vs. 523 T), kidney renal papillary cell carcinoma (KIRP, 607 N vs. 286 T), acute myeloid leukemia (LAML, 707 N vs. 173 T), low grade glioma (LGG, 207 N vs. 518 T), liver hepatocellular carcinoma (LIHC, 160 N vs. 369 T), lung adenocarcinoma (LUAD, 347 N vs. 483 T), lung squamous cell carcinoma (LUSC, 338 N vs. 486 T), ovarian carcinoma (OV, 88 N vs. 426 T), pancreatic adenocarcinoma (PAAD, 171 N vs. 179 T), prostate adenocarcinoma (PRAD, 1521 N vs. 492 T), rectum adenocarcinoma (READ, 318 N vs. 92 T), thyroid carcinoma (THCA, 337 N vs. 512 T), uterine corpus rndometrial carcinoma (UCEC, 91 N vs. 174 T) and uterine carcinosarcoma (USC, 78 N vs. 57 T). [file 13046_2021_2096_MOESM2_ESM.zip › Supplementary Figure2-1.jpg]

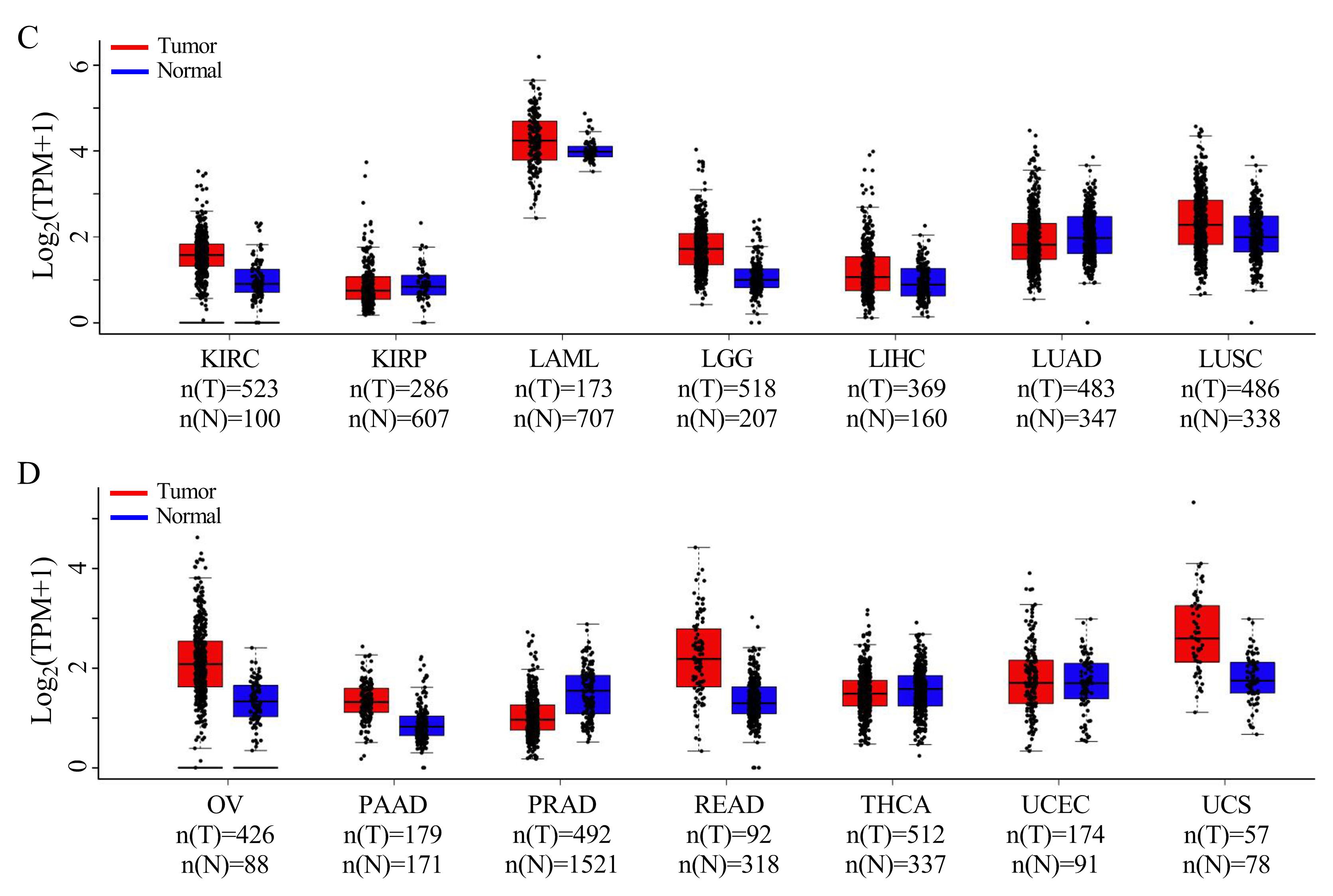

Supplement: Supplementary file 2 — Additional file 2: Suppl. Fig. 2 Pan-cancer analysis based on GEPIA database reveals the expression landscape of LINC00022 in human cancers. The comprehensive cancer database GEPIA was employed to investigate the expression landscape of LINC00022 in 28 types of cancer. (A) LINC00022 was significantly increased in cervical squamous carcinoma (CESC, 13 N vs. 306 T), lymphoid neoplasm diffused large B-cell lymphoma (DLBC, 337 N vs. 47 T), glioblastoma (GBM, 207 N vs. 163 T), skin cutaneous melanoma (SKCM, 558 N vs. 461 T), stomach adenocarcinoma (STAD, 211 N vs. 408 T) and thymoma (THYM, 339 N vs. 118 T), while decreased in testicular germ cell tumor (TGCT, 165 N vs. 137 T). (B-D) LINC00022 expression showed no significant differences between tumor samples and normal samples in adrenocortical carcinoma (ACC, 128 N vs. 77 T), bladder urothelial carcinoma (BLCA, 28 N vs. 404 T), breast cancer (BRCA, 291 N vs. 1085 T), cholangio carcinoma (CHOL, 9 N vs. 36 T), colon adenocarcinoma (COAD, 349 N vs. 275 T), head and neck squamous cell carcinoma (HNSC, 44 N vs. 519 T), kidney chromophobe (KICH, 53 N vs. 66 T), kidney renal clear cell carcinoma (KIRC, 100 N vs. 523 T), kidney renal papillary cell carcinoma (KIRP, 607 N vs. 286 T), acute myeloid leukemia (LAML, 707 N vs. 173 T), low grade glioma (LGG, 207 N vs. 518 T), liver hepatocellular carcinoma (LIHC, 160 N vs. 369 T), lung adenocarcinoma (LUAD, 347 N vs. 483 T), lung squamous cell carcinoma (LUSC, 338 N vs. 486 T), ovarian carcinoma (OV, 88 N vs. 426 T), pancreatic adenocarcinoma (PAAD, 171 N vs. 179 T), prostate adenocarcinoma (PRAD, 1521 N vs. 492 T), rectum adenocarcinoma (READ, 318 N vs. 92 T), thyroid carcinoma (THCA, 337 N vs. 512 T), uterine corpus rndometrial carcinoma (UCEC, 91 N vs. 174 T) and uterine carcinosarcoma (USC, 78 N vs. 57 T). [file 13046_2021_2096_MOESM2_ESM.zip › Supplementary Figure2-2.jpg]

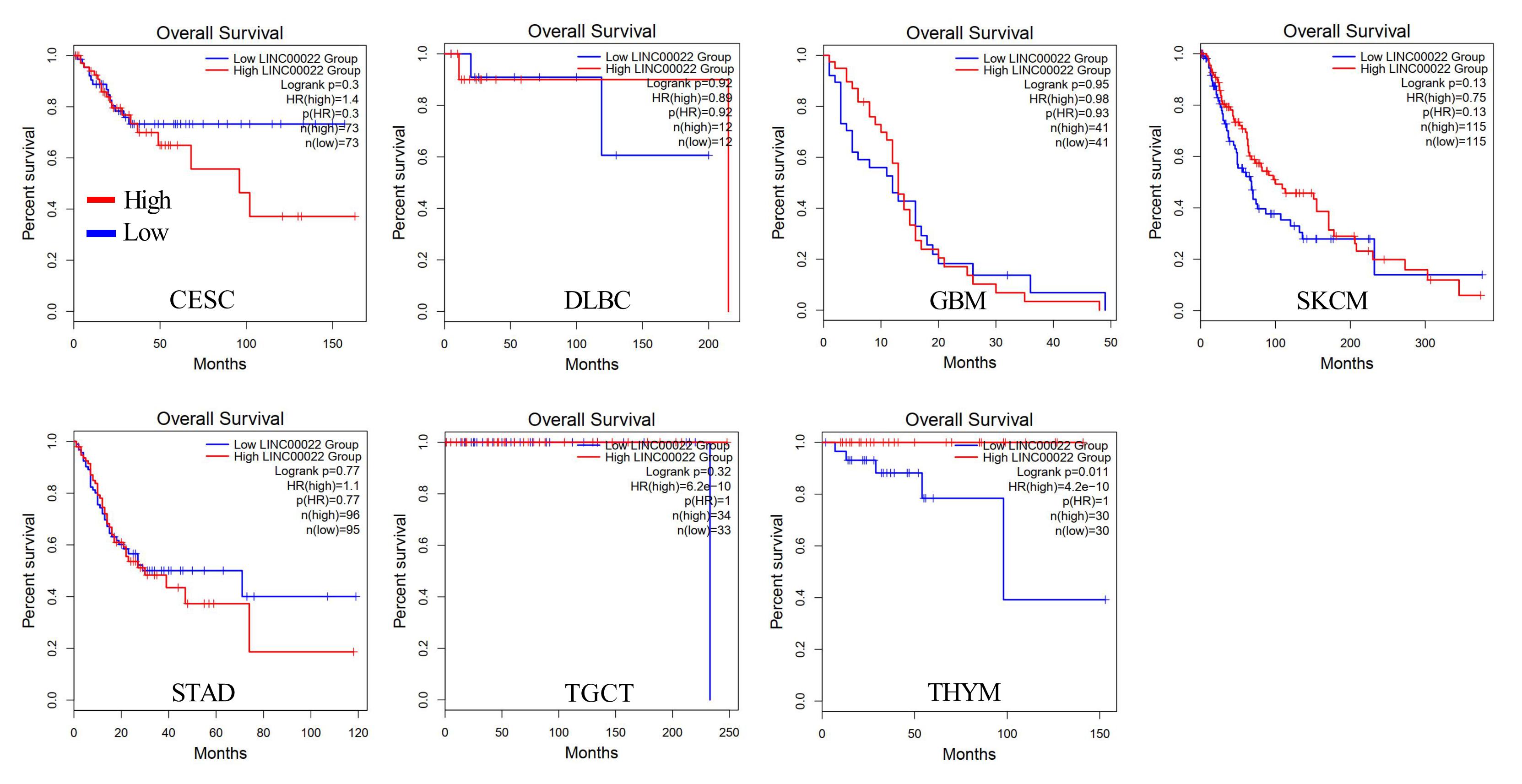

Supplement: Supplementary file 3 — Additional file 3: Suppl. Fig. 3 Prognostic analysis of LINC00022 in CESC, DLBC, GBM, SKCM, STAD, TGCT and THYM based on GEPIA database. GEPIA was utilized to analyze the prognostic significance of LINC00022 in seven types of cancer with dysregulated expression. LINC00022 expression was not significantly associated with OS in patients with CESC, DLBC, GBM, SKCM, STAD and TGCT, except for THYM. [file 13046_2021_2096_MOESM3_ESM.jpg]

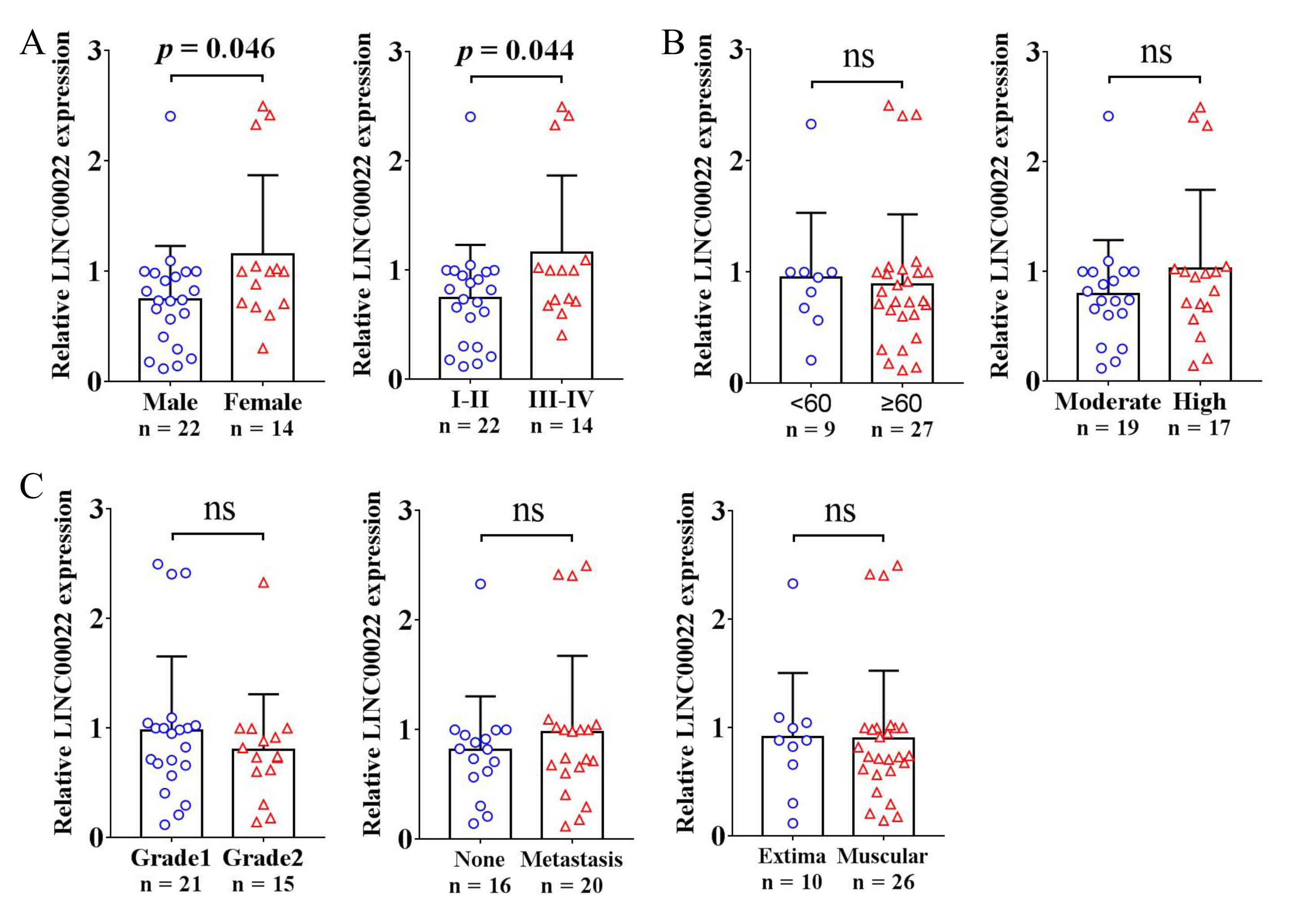

Supplement: Supplementary file 4 — Additional file 4: Suppl. Fig. 4 Relationships between LINC00022 expression and clinical characteristics of ESCC patients in our study cohort. (A) The expression of LINC00022 in tumor tissues of female patients was obviously higher than that of male patients, p = 0.046. (B) LINC00022 expression in tumor tissues of stage III-IV patients was significantly higher than that in tumor tissues of stage I-II patients, p = 0.044. (B-C) No significant correlation was found between the expression of LINC00022 and age, differentiation, grade, lymph node metastasis or depth of invasion. [file 13046_2021_2096_MOESM4_ESM.jpg]

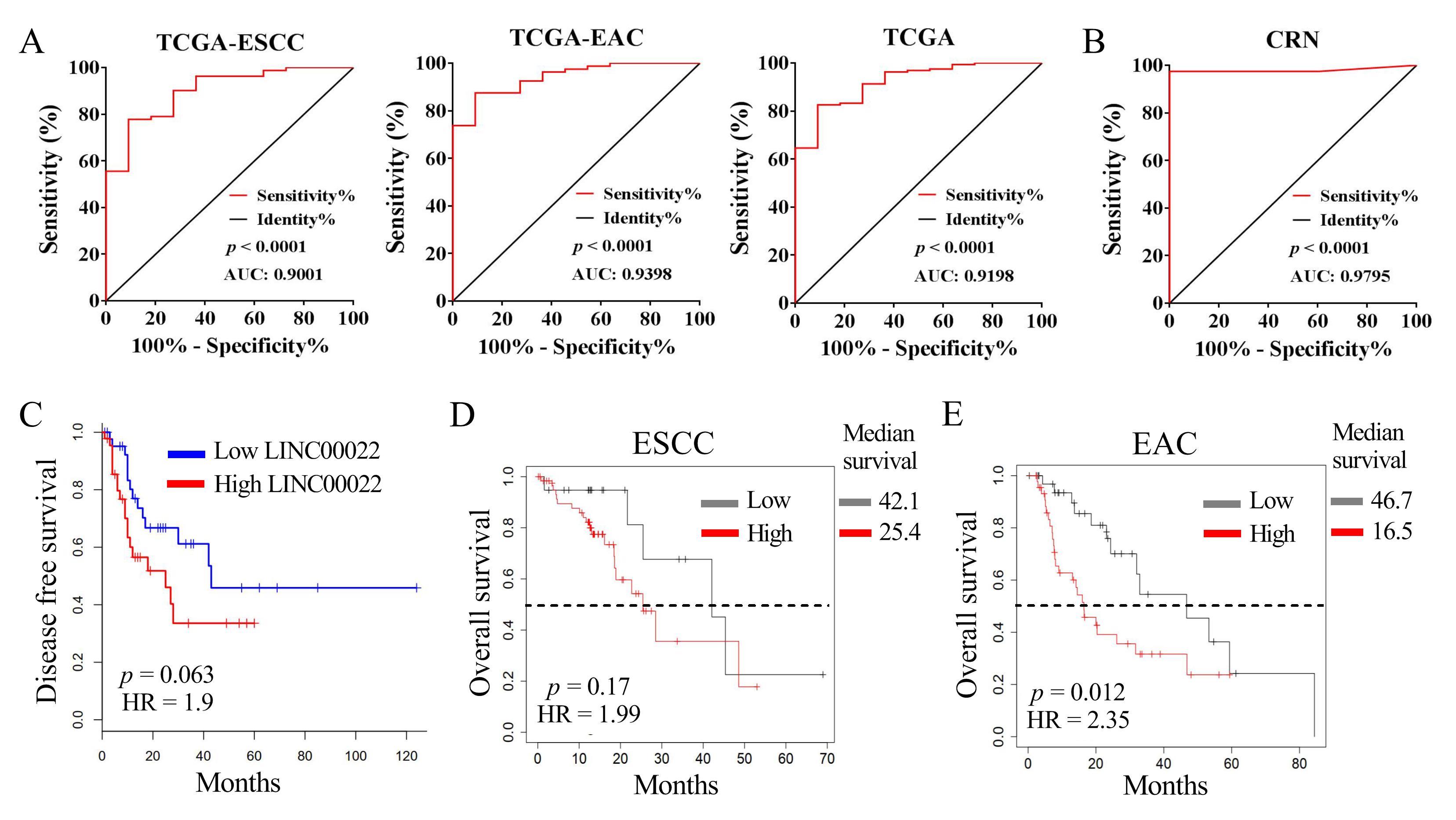

Supplement: Supplementary file 5 — Additional file 5: Suppl. Fig. 5 The diagnostic and prognostic values of LINC00022 in various ESCC cohorts. (A) ROC analysis suggested the high diagnostic value of LINC00022 in the TCGA-ESCC and TCGA-EAC cohorts (AUC > 0.9 and p < 0.0001). (B) The AUC value of LINC00022 in CRN cohort was as high as 0.9795 (p < 0.0001). (C) The prognostic significance of LINC00022 in GEPIA cohort was determined by the Kaplan-Meier method. Elevated LINC00022 expression indicated worse patient DFS. (D-E) Kaplan-Meier analysis from TCGA-ESCC and TCGA-EAC cohorts showed that patients with higher LINC00022 expression had a shorter median OS. [file 13046_2021_2096_MOESM5_ESM.jpg]

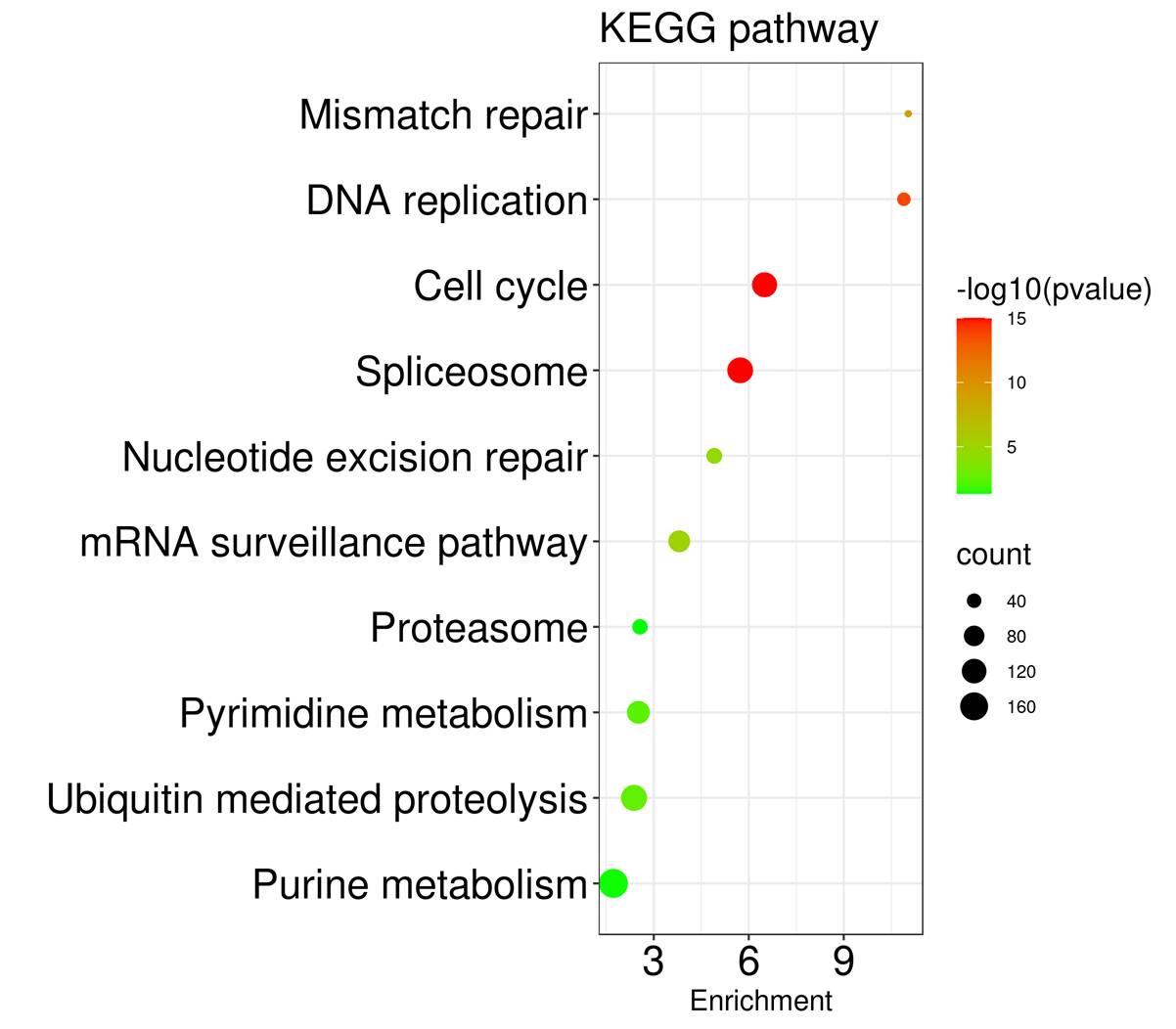

Supplement: Supplementary file 6 — Additional file 6: Suppl. Fig. 6 KEGG pathway analysis reveals the crucial role of LINC00022 in ESCC. 1000 genes associated with LINC00022 in ESCC tumors was obtained from GEPIA database and subjected to KEGG pathway analysis. The enrichment result was shown as a bubble chart. KEGG analysis depicted that LINC00022 may be mainly involved in mismatch repair, DNA replication, cell cycle, spliceosome, nucleotide excision repair, mRNA surveillance pathway, proteasome, pyrimidine metabolism, ubiquitin mediated proteolysis, and purine metabolism. [file 13046_2021_2096_MOESM6_ESM.jpg]

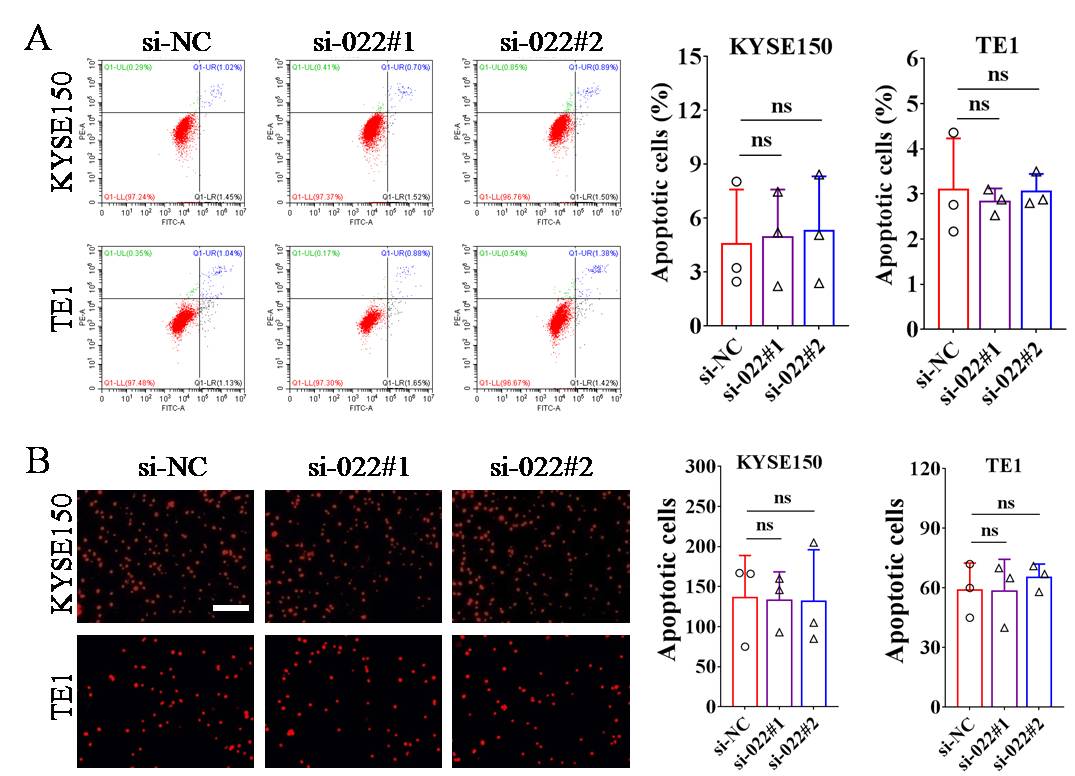

Supplement: Supplementary file 7 — Additional file 7: Suppl. Fig. 7 Knockdown of LINC00022 has no significant impact on apoptosis of ESCC cells. (A) Annexin V-FITC/PI double staining and flow cytometry was used to detect apoptosis of KYSE150 and TE1 cells following LINC00022 silence. (B) PI-labeling combined with fluorescent microscope revealed that LINC00022 did not contribute to cell apoptosis of ESCC. [file 13046_2021_2096_MOESM7_ESM.jpg]

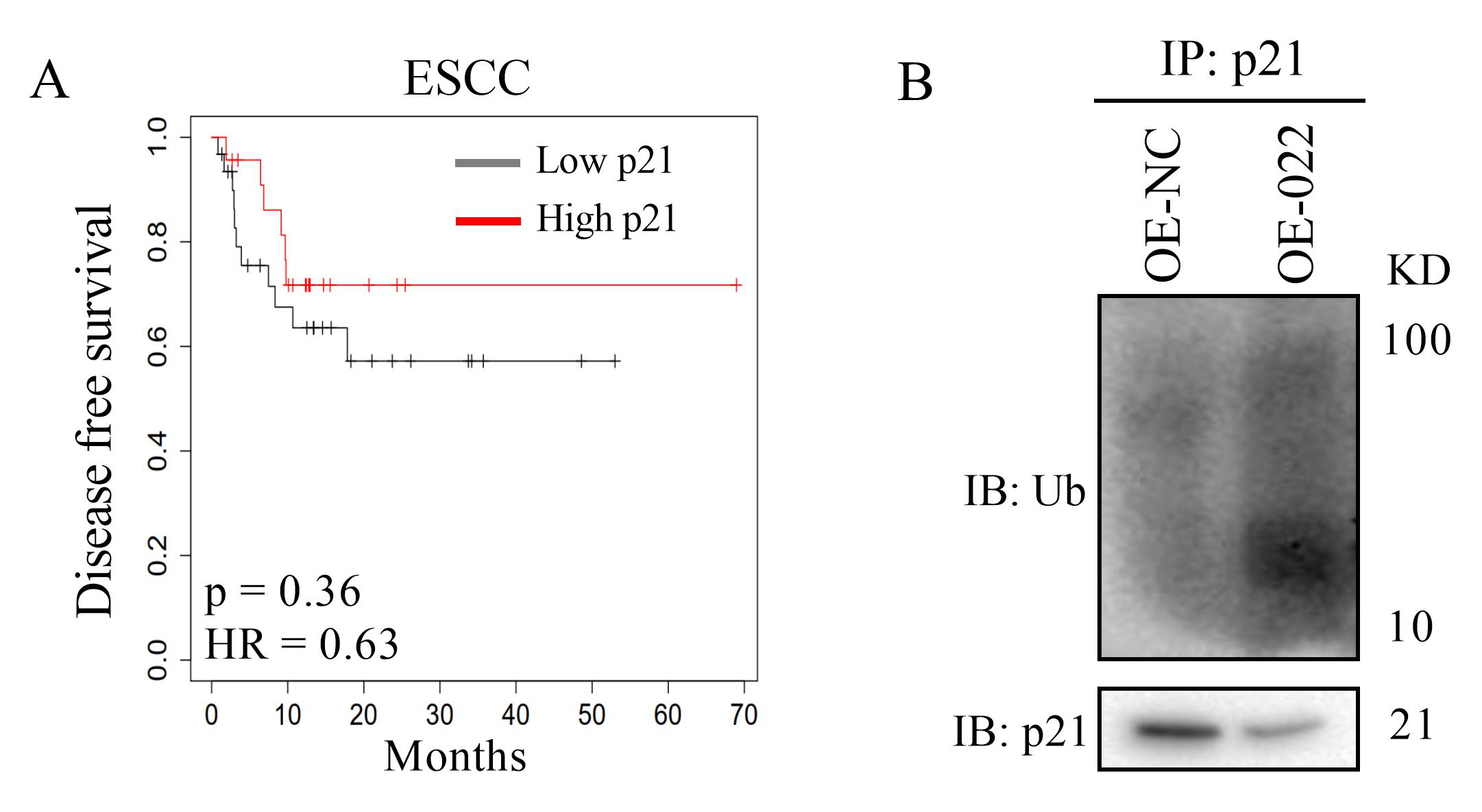

Supplement: Supplementary file 8 — Additional file 8: Suppl. Fig. 8 The protein expression and ubiquitination level of p21 in LINC00022-augmented tumors. (A) The prognostic significance of p21 in TCGA-ESCC cohort was analyzed by the Kaplan-Meier method. Elevated p21 expression indicated better patient DFS. (B) Co-IP and Western blot were performed to examine the protein level and ubiquitination of p21 in OE-NC and OE-022 tumors derived from the in vivo experiment. [file 13046_2021_2096_MOESM8_ESM.jpg]

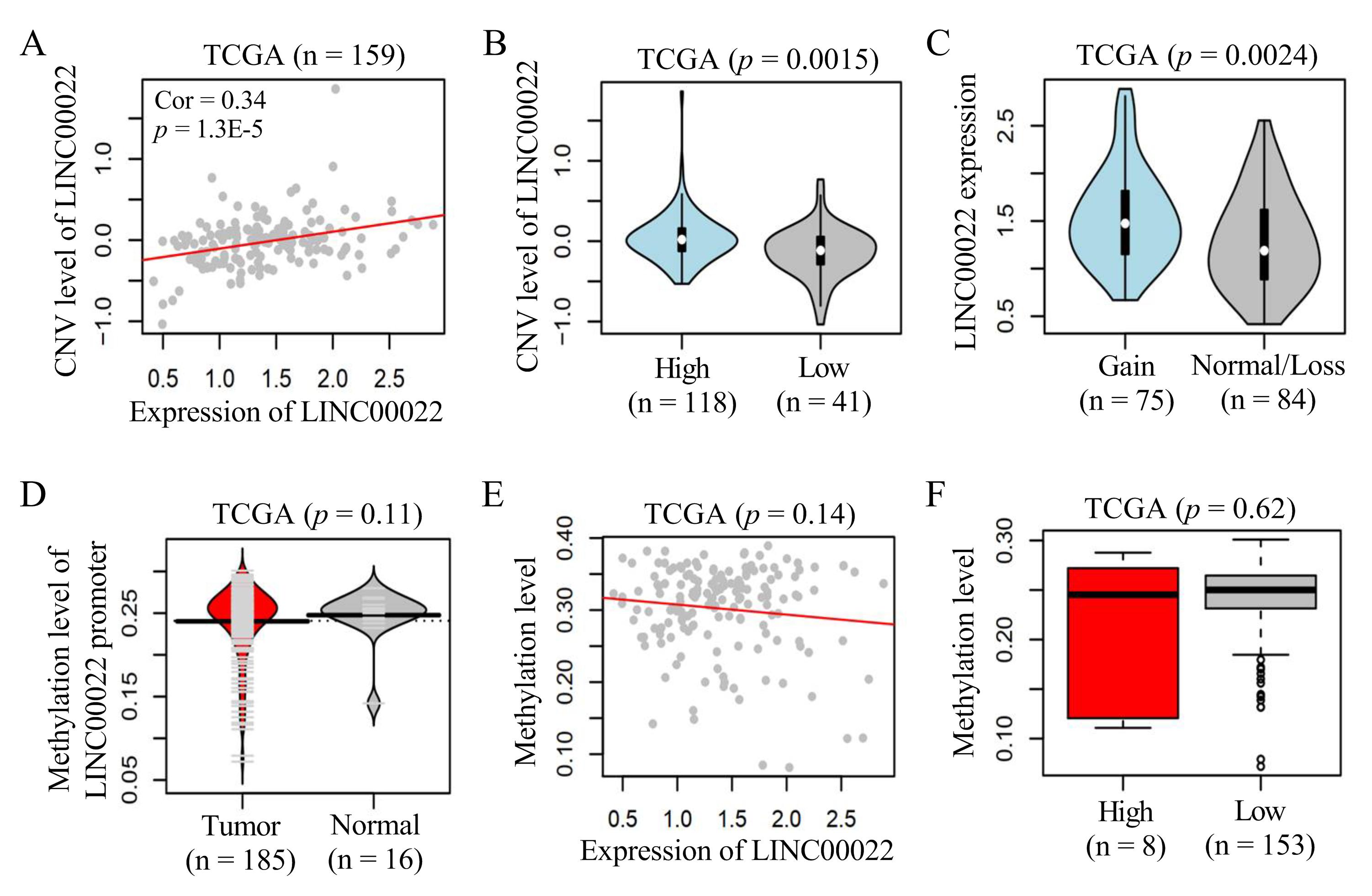

Supplement: Supplementary file 9 — Additional file 9: Suppl. Fig. 9 Copy number variation (CNV), rather than DNA methylation, correlates with LINC00022 expression in ESCA. (A) The genome CNV level of LINC00022 was positively correlated with its expression in 159 cases of ESCA samples from TCGA, Cor = 0.35 and p = 1.3E-5. (B) Tumor samples with high expression of LINC00022 had higher CNV level. The 159 cases of tumor samples were divided into High and Low groups according to the upper 95 quantile of LINC00022 expression in normal samples as the threshold, p = 0.0015. (C) The gene amplified tumor samples showed higher expression of LINC00022. The 159 cases of tumor samples were divided into Gain and Normal/Loss groups according to logratio value 0 of copy number as the threshold. (D) The methylation level of LINC00022 promoter showed no significant differences between tumor and normal samples in ESCA. (E-F) The methylation level of LINC00022 promoter was not significantly associated with its expression in ESCA samples from TCGA. [file 13046_2021_2096_MOESM9_ESM.jpg]

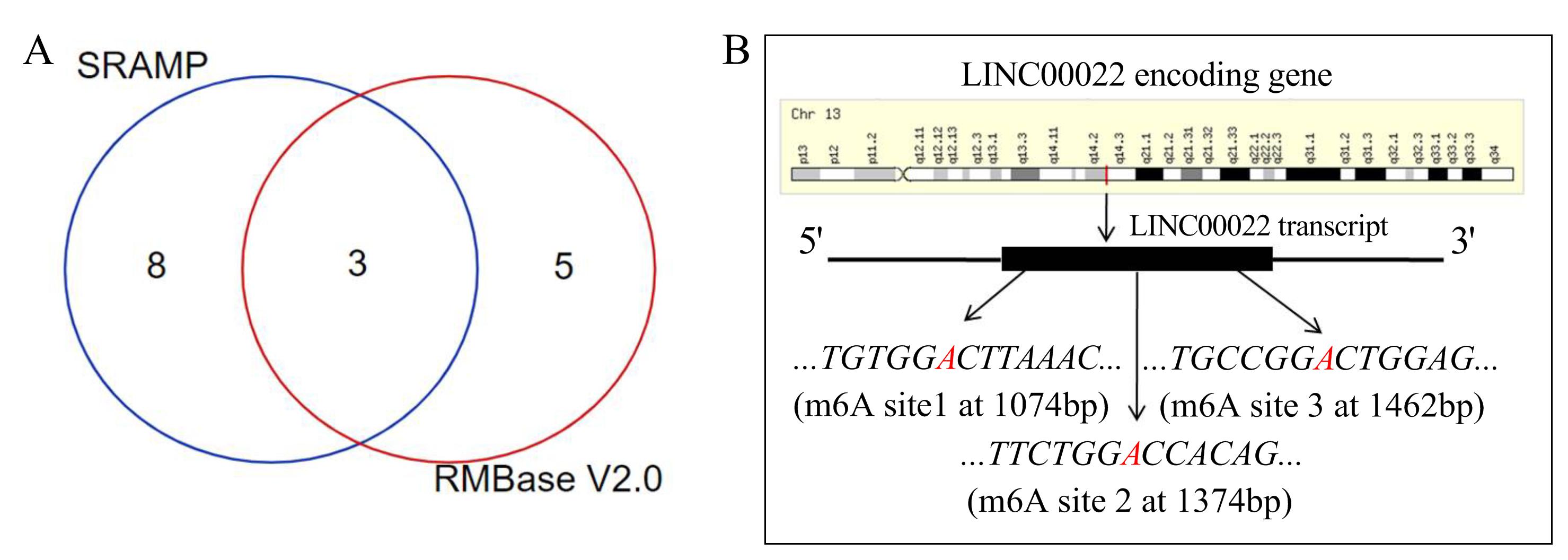

Supplement: Supplementary file 10 — Additional file 10: Suppl. Fig. 10 LINC00022 transcript contains many potential m6A modification sites. (A) The algorithm of software SRAMP and RMBase V2.0 analyzed 11 and 8 highly reliable m6A sites on the LINC00022 transcript, respectively. (B) Three overlapped m6A loci with high confidence were located at 1074 bp, 1374 bp, 1462 bp from 5′-end on the LINC00022 transcript. [file 13046_2021_2096_MOESM10_ESM.jpg]

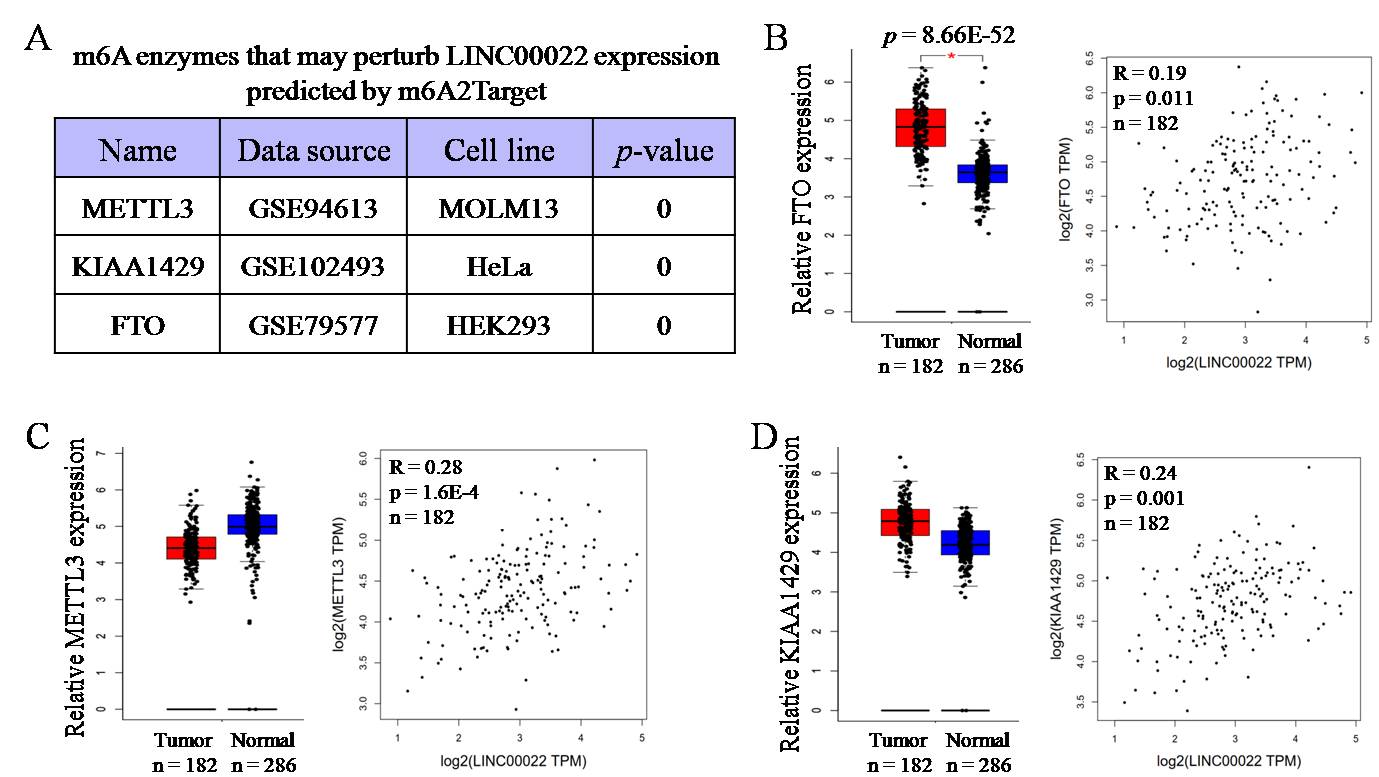

Supplement: Supplementary file 11 — Additional file 11: Suppl. Fig. 11 FTO is up-regulated in ESCC and positively correlated with LINC00022 expression. (A) The online tool m6A2Target, based on sequencing validation data, was used to analyze m6A modification enzymes that may perturb LINC00022 expression. (B-D) The expression of FTO, METTL3 and KIAA1429 in ESCC and their correlation with LINC00022 was analyzed by the GEPIA database. [file 13046_2021_2096_MOESM11_ESM.jpg]

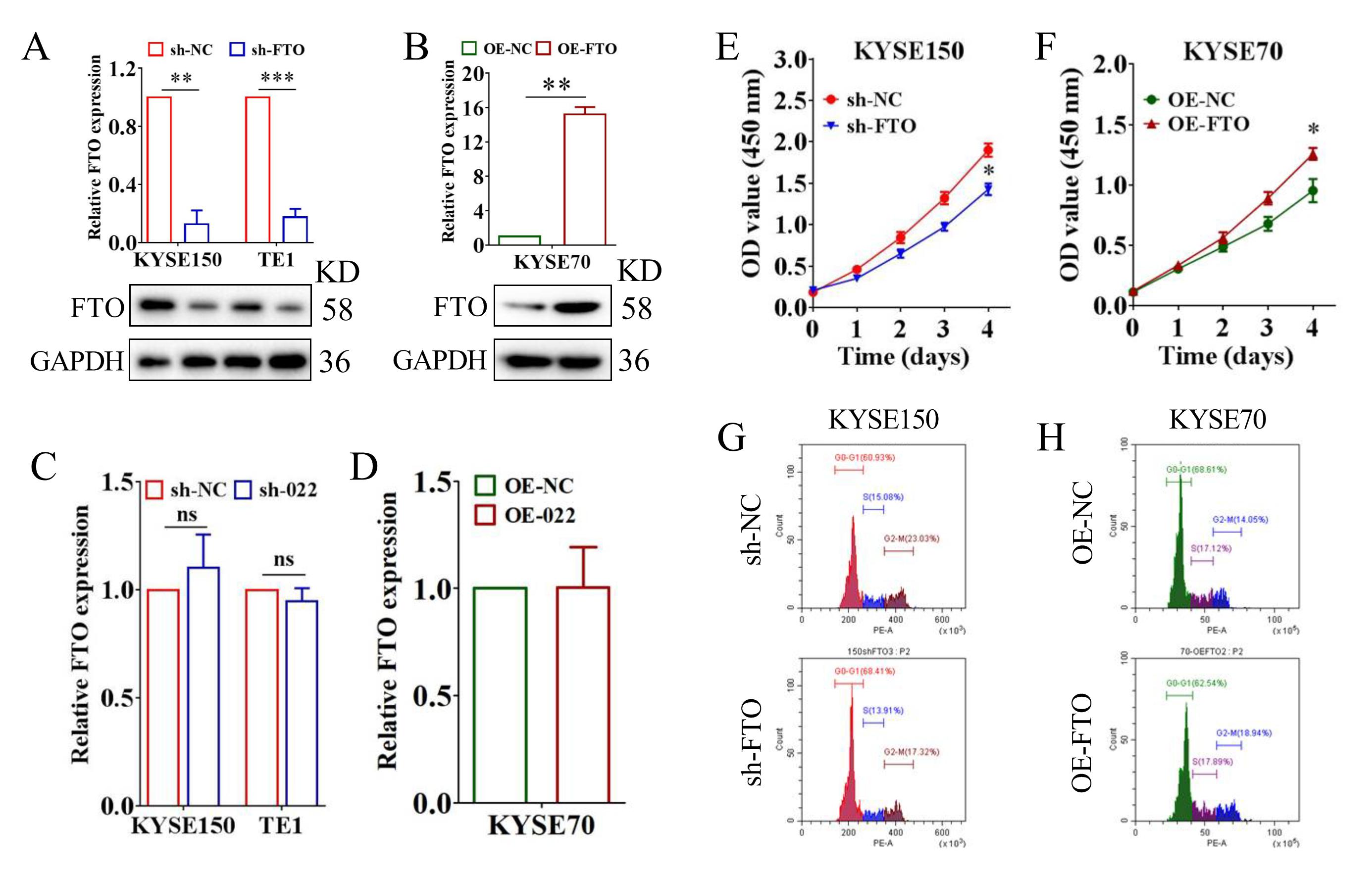

Supplement: Supplementary file 12 — Additional file 12: Suppl. Fig. 12 FTO promotes ESCC proliferation and cell-cycle progression. (A) The knockdown efficiencies of FTO mediated by recombinant lentivirus were validated at the mRNA and protein levels in KYSE150 and TE1 cells by qRT-PCR (upper panel, **p < 0.01; ***p < 0.001) and Western blot (nether panel), respectively. (B) The over-expression efficiencies of FTO mediated by recombinant lentivirus were validated at the mRNA and protein levels in KYSE70 cells by qRT-PCR (upper panel, **p < 0.01) and Western blot (nether panel), respectively. (C-D) Both the ablation and over-expression of LINC00022 had no significant effect on the expression on FTO in ESCC cells as depicted by qRT-PCR. (E-F) CCK-8 assay was employed to evaluate cell viability of KYSE150 with FTO knockdown (A), and KYSE70 with FTO over-expression (B), *p < 0.05. (G-H) Cell cycle phase distribution of KYSE150 or KYSE70 following FTO knockdown or over-expression was detected by PI-staining flow cytometry. [file 13046_2021_2096_MOESM12_ESM.jpg]

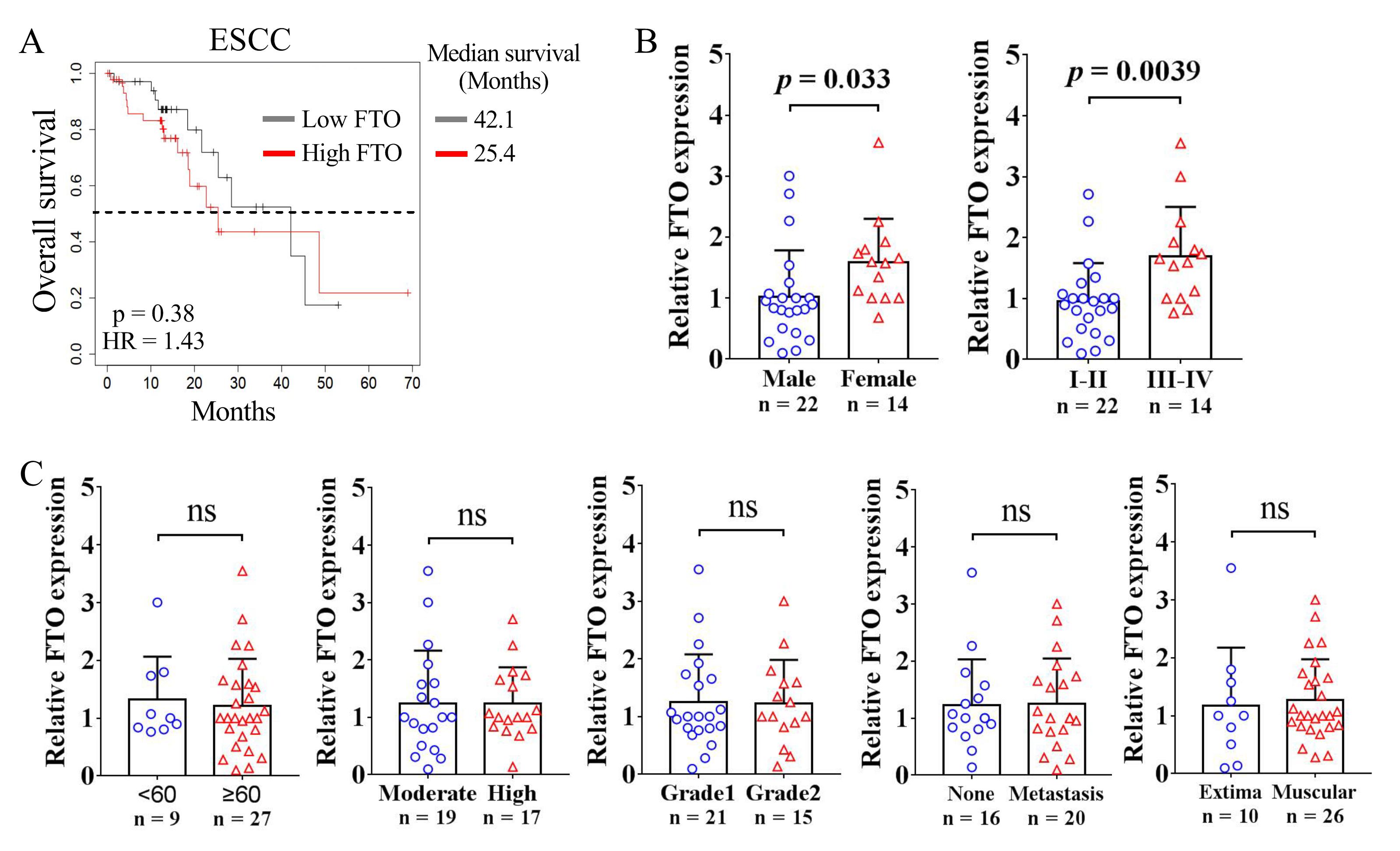

Supplement: Supplementary file 13 — Additional file 13: Suppl. Fig. 13 Relationships between FTO expression and clinical characteristics of ESCC patients in our study cohort. (A) Kaplan-Meier analysis from TCGA-ESCC cohort showed that patients with higher FTO expression had a shorter median OS. (B) The expression of FTO in tumor tissues of female patients was obviously higher than that of male patients, p = 0.033. FTO expression in tumor tissues of stage III-IV patients was significantly higher than that in tumor tissues of stage I-II patients, p = 0.0039. (C) No significant correlation was found between the expression of FTO and age, differentiation, grade, lymph node metastasis or depth of invasion. [file 13046_2021_2096_MOESM13_ESM.jpg]

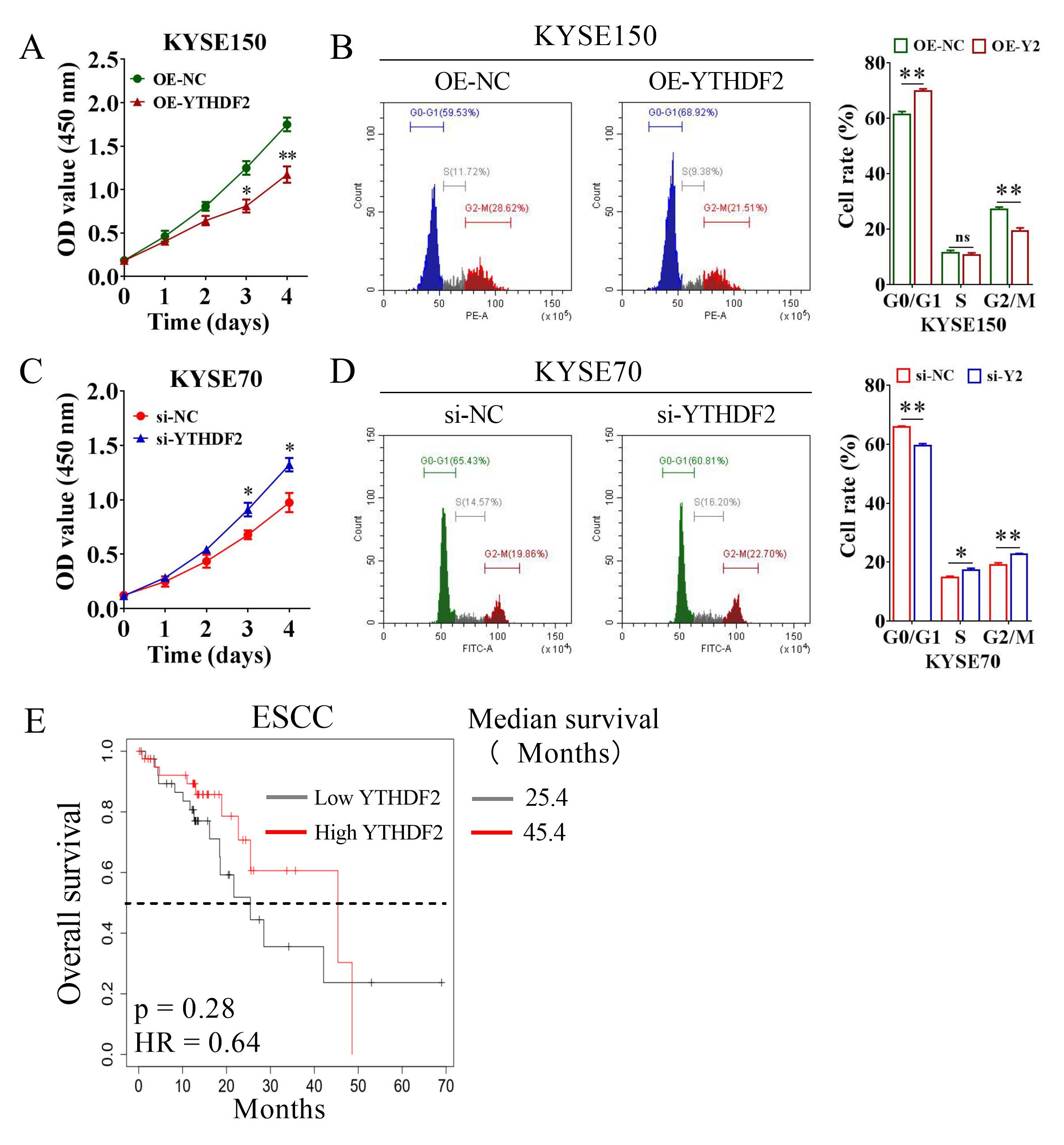

Supplement: Supplementary file 14 — Additional file 14: Suppl. Fig. 14 YTHDF2 suppresses ESCC proliferation and cell-cycle progression. (A) CCK-8 experiment was performed to evaluate cell proliferation of KYSE150 with YTHDF2 over-expression, **p < 0.01. (B) Flow cytometry was utilized to examine cell cycle changes of KYSE150 after over-expression of YTHDF2, **p < 0.01. (C) Knockdown of YTHDF2 enhanced cell proliferation of KYSE70 cells, *p < 0.05. (D) Silencing of YTHDF2 promoted cell cycle progression of KYSE70 cells, *p < 0.05; **p < 0.01. (E) The prognostic significance of YTHDF2 in TCGA-ESCC cohort was analyzed by the Kaplan-Meier method. Increased YTHDF2 expression indicated better patient OS. [file 13046_2021_2096_MOESM14_ESM.jpg]

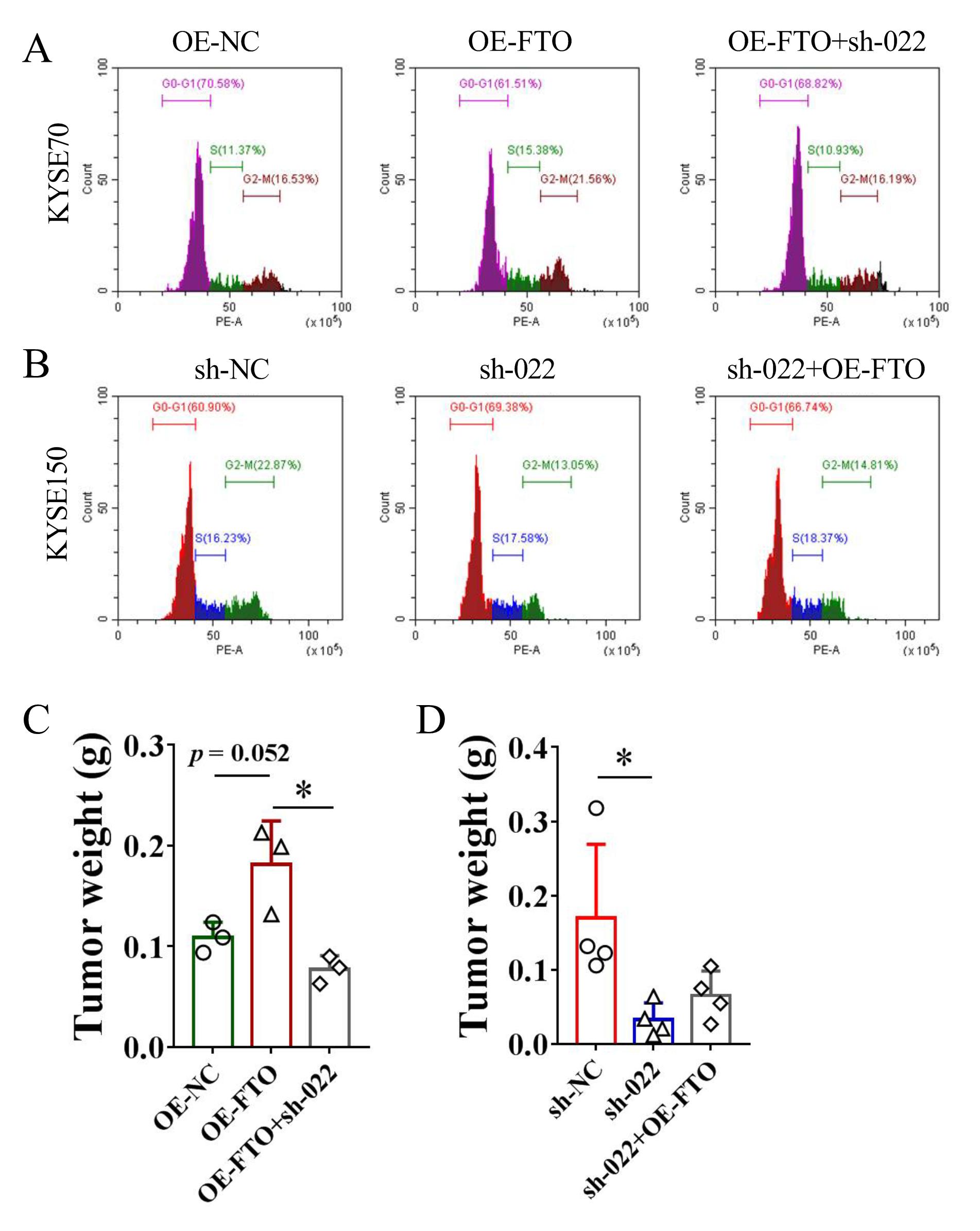

Supplement: Supplementary file 15 — Additional file 15: Suppl. Fig. 15 FTO/LINC00022 axis regulates cell-cycle and tumorigenesis of ESCC. (A-B) PI-labeling staining combined with flow cytometry revealed the role of FTO/LINC00022 axis in ESCC cell-cycle progression. (C) Knockdown of LINC00022 fully rescued the growth promotion effect caused by ectopic FTO expression on KYSE70 cells in nude mice as indicated by tumor weight (n = 3), *p < 0.05. (D) Over-expression of FTO partially attenuated the inhibition of subcutaneous tumorigenicity induced by LINC00022 knockdown on KYSE150 cells in nude mice as indicated by tumor weight (n = 4), *p < 0.05 [file 13046_2021_2096_MOESM15_ESM.jpg]
